# Natural variant frequencies across domains from different sarcomere proteins cross-correlate to identify inter-protein contacts associated with cardiac muscle function and disease

**DOI:** 10.1186/s43556-021-00056-x

**Published:** 2021-11-15

**Authors:** Thomas P. Burghardt

**Affiliations:** Department of Biochemistry and Molecular Biology, Mayo Clinic Rochester, 200 First St. SW, Rochester, MN 55905 USA

**Keywords:** Single nucleotide variants, Cardiac muscle inheritable disease, 2D Correlation genetics, Interprotein coordinated domains, Serial founder effect, Population genetic divergence proxy

## Abstract

Coordinated sarcomere proteins produce contraction force for muscle shortening. In human ventriculum they include the cardiac myosin motor (βmys), repetitively converting ATP free energy into work, and myosin binding protein C (MYBPC3) that in complex with βmys is regulatory. Single nucleotide variants (SNVs) causing hereditary heart diseases frequently target this protein pair. The βmys/MYBPC3 complex models a regulated motor and is used here to study how the proteins couple. SNVs in βmys or MYBPC3 survey human populations worldwide. Their protein expression modifies domain structure affecting phenotype and pathogenicity outcomes. When the SNV modified domain locates to inter-protein contacts it could affect complex coordination. Domains involved, one in βmys the other in MYBPC3, form coordinated domains (co-domains). Co-domain bilateral structure implies the possibility for a shared impact from SNV modification in either domain suggesting a correlated response to a common perturbation could identify their location. Genetic divergence over human populations is proposed to perturb SNV probability coupling that is detected by cross-correlation in 2D correlation genetics (2D-CG). SNV probability data and 2D-CG identify three critical sites, two in MYBPC3 with links to several domains across the βmys motor, and, one in βmys with links to the MYBPC3 regulatory domain. MYBPC3 sites are hinges sterically enabling regulatory interactions with βmys. The βmys site is the actin binding C-loop (residues 359-377). The C-loop is a trigger for actin-activated myosin ATPase and a contraction velocity modulator. Co-domain identification implies their spatial proximity suggesting a novel approach for in vivo protein complex structure determination.

**Supplementary Information:**

The online version contains supplementary material available at 10.1186/s43556-021-00056-x.

## Introduction

**Fig. 1.**
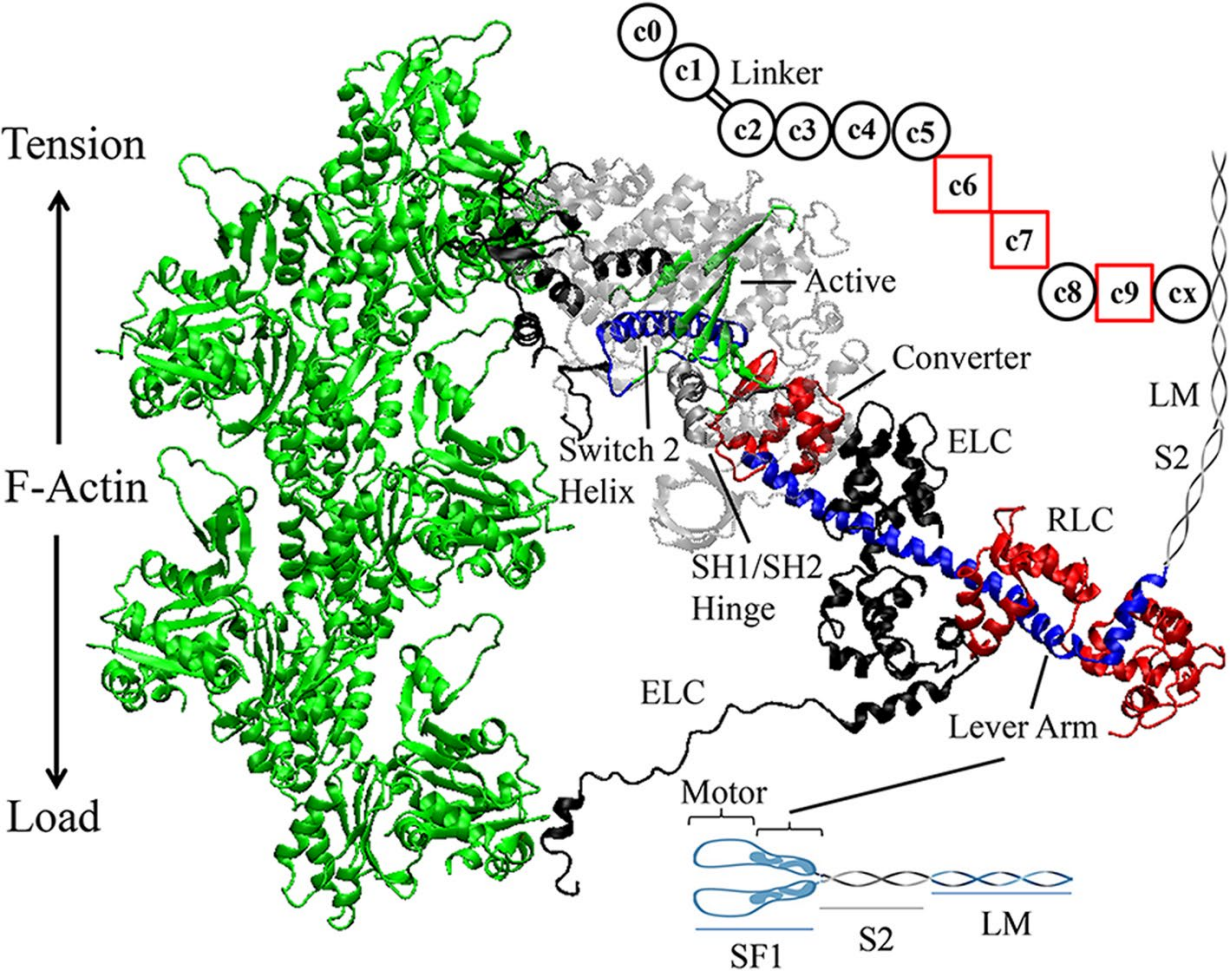
Myosin and MYBPC3 structures in the sarcomere. Myosin dimer model (bottom right) has two subfragment 1 (SF1), subfragment 2 (S2), and light meromyosin (LM) polypeptides. SF1 crystal structure has a motor domain and a lever arm (blue) with bound light chains ELC (black) and RLC (red). The motor domain contains several binding sites for actin, the active site binding ATP (green), the switch 2 helix (blue), SH1/SH2 hinge (silver), and converter domain (red). MYBPC3 model (upper right) has multiple domains shown schematically in black circles or red squares indicating immunoglobulin-like or fibronectin-like domains with CX binding myosin LM, N-terminal C0-C2 maintaining transient interactions with F-actin (green) and myosin, and the linker containing regulatory phosphorylation sites. Tension and load vectors indicate direction of force generated by myosin and opposed by F-actin, respectively. F-actin and SF1 homology models were obtained as described [1]

Muscle proteins in the sarcomere assemble into machinery needed to generate and regulate muscle contraction by their coordinated action. In the human heart ventriculum, cardiac ventricular myosin (βmys) is the molecular motor repetitively converting ATP free energy into work. Three genes, MYH7 (heavy chain), MYL3 (essential light chain or ELC), and MYL2 (regulatory light chain or RLC) encode βmys (Fig. 1). The heavy chain has a 140 kDa N-terminal globular motor domain (subfragment 1, SF1, or myosin head) and an extended α-helical tail domain forming subfragment 2 (S2) and light meromyosin (LM). LM domains form dimers and self-assemble into thick filaments with SF1 and S2 projecting outward from the core in a helical array [2]. Thick filaments interdigitate with actin thin filaments (filamentous or F-actin) in the sarcomere where βmys motors cyclically interact with F-actin [3]. SF1 binds to an actin filament and rotates the lever arm generating torque and tension on F-actin [4, 5]. The ELC N-terminus also binds actin generating force and ending the power stroke [6]. Relative movement of motor subdomains is the structural manifestation of the energy conversion wherein ATP hydrolysis at the active site modulates actin affinity at actin binding sites, induces small active site translations amplified into larger structural changes at the switch 2 helix and SH1/SH2 hinge, and the latter converted by the converter domain into the lever arm swing.

**Fig. 2.**
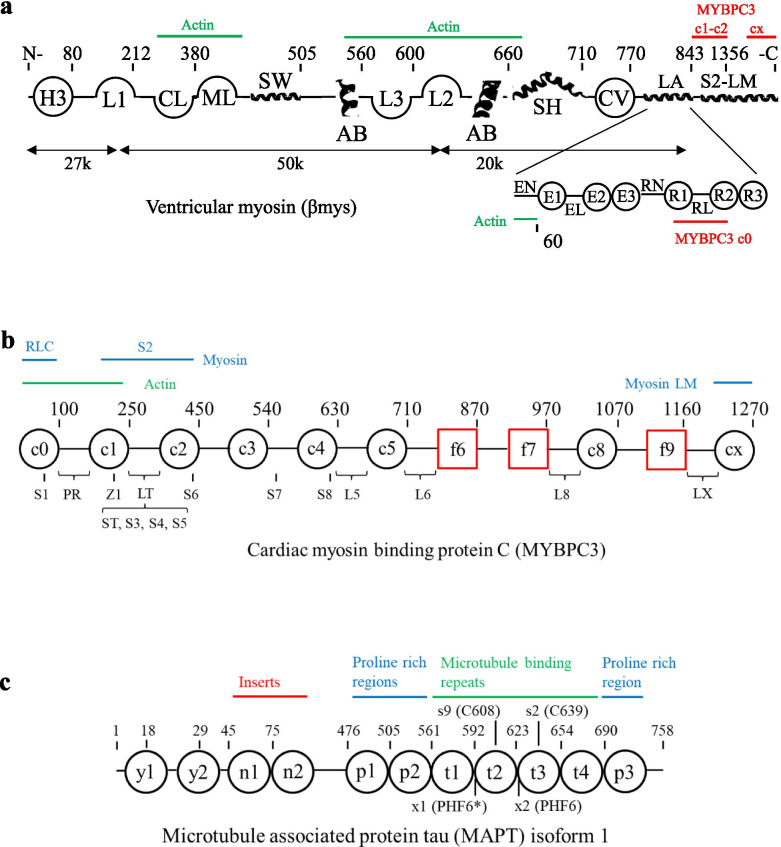
Linearized diagrams for βmys, MYBPC3, and microtubule-associated protein tau (MAPT). Diagrams identify most domains defined in Supplementary Tables S1 and S4 and approximate residue numbering. **a** The βmys diagram has a heavy chain (H3 through LM), ELC (EN through E3), and RLC (RN through R3). Heavy chain diagram does not indicate the active site (AC), Omecamtiv Mecarbil binding site (OM), and mesa (ME) because they occupy multiple regions in the linear representation. MYBPC3 and actin binding sites are indicated in red and green above the heavy chain and below the light chains. **b** MYBPC3 has Ig-like (black circles) and fibronectin-like domains (red squares). Serine phosphorylation sites, S1 (Ser47), ST (Ser273), S3 (Ser282), S4 (Ser302), and S5-S7 and a threonine phosphorylation site (S8) are indicated below the chain. Domain linkers of interest include the proline rich linker (PR) and LT containing a regulatory site. Z1 is a zinc binding site. Myosin RLC, S2, LM, and actin binding sites on MYBPC3 are indicated above the linearized model. **c** The MAPT diagram shows the largest isoform (isoform 1). Sites or domains identified are tyrosine phosphorylation sites (y1 and y2), N-terminal inserts (n1 and n2), proline rich regions (p1-p3), microtubule binding repeats (t1-t4), cysteines C608 and C639 (s9 and s2), and hexapeptide motifs PHF6* (VQIINK at x1) and PHF6 (VQIVYK at x2)

Cardiac myosin binding protein C (MYBPC3), denoting either the gene or expressed protein depending on context, localizes to the C-zone in the cardiac muscle sarcomere where it associates with one in three βmys motors [7]. It regulates energy conversion and shortening velocity by transient N-terminus binding to actin or myosin with the C-terminus fixed to the myosin thick filament [8]. MYBPC3 has 8 immunoglobulin-like and 3 fibronectin-like domains in a linear array (Fig. 2). Peptide linker 2 (LT), in the N-terminus linking C1 and C2, contains phosphorylation sites participating in contractile regulation by modulating myosin activity [9–11] and calcium sensitivity [12]. MYBPC3 N-terminus associates alternatively with actin [13], the myosin motor [14], and myosin thick filament [13] while C-terminus binds LM in the thick filament with domains C8–CX [15]. Two hinge points in the linear molecule, at LT and near C5, were identified [16]. A plausible third hinge near the C-terminus would facilitate known multiple protein interactions with βmys. The βmys and MYBPC3 bimolecular complex, βmys/MYBPC3, is a model for the contractile system and for studying interactions coupling them.

Non-synonymous single nucleotide variants (SNVs) change protein sequences in βmys or MYBPC3. A variant sequence modifies protein domain structure and function affecting phenotype and pathogenicity outcomes. When a SNV modified domain locates to the point of contact in the βmys/MYBPC3 complex it changes complex interoperability and the domains involved, one in βmys the other in MYBPC3, are inter-protein coordinated functional domains (co-domains). The bilateral nature of the co-domain implies a joint impact from modification at one end or the other and a correlated response to a common perturbation should identify their presence. Moreover, βmys and MYBPC3 SNVs have pathogenicities that correlate with human populations [17] indicating that cardiac muscle physiology integrates βmys/MYBPC3 complex functionality with the wider system involving genetic background [18]. It is proposed to leverage genetic background influence to identify co-domains.

Human population genetic divergence decreases linearly with increasing human migration distance over the earth’s surface from a single origin in Africa [19]. Migration distance is then a proxy for genetic differentiation providing the means to use genetic background, in an ordered sequence of human populations, to perturb SNV probability. 2-dimensional correlation genetics (2D-CG) is introduced where systematic genetic divergence over human populations is the perturbation coupling SNV probability across co-domains detected by cross-correlation.

2D-CG is analogous to 2D correlation spectroscopy [20] wherein βmys and MYBPC3 functional domains replace the two spectral frequencies near resonance, SNV probability products mimic resonance absorption intensity, and human population genetic differentiation provides the arithmetically indexed perturbation. In concept, 2D-CG is also analogous to 2D-NMR. RF pulse magnetization transfer is the perturbation cross-correlating chemical shifts of specific molecular nuclei in 2D-NMR. Here chemical physics laws relate spatial distance separating cross-correlated nuclei with signal intensity giving a structural distance constraint [21].

Physical characteristics for each SNV including substituted residue location in the protein functional domain, side chain substitution, substitution frequency, and human population group are unique and consistently recorded in the database from one or more data providers. Implicated characteristics for each SNV including phenotype and pathology outcomes with identical physical characteristics from one or more providers are consistently or inconsistently identified among providers, or, unknown. The consistent subset of the data trains and validates a feed-forward neural network model of the contraction mechanism. The full database is completed by replacing inconsistent or unknown outcomes with the model derived outcomes then interpreted probabilistically with a discrete Bayes network to give the SNV probability for a functional domain location given pathogenicity and human population. The co-domains have member domain SNV probabilities that cross-correlate over human populations for given pathogenicity. They identify three critical regulatory sites, two in MYBPC3 with links to several domains across the βmys motor, and, one in βmys with links to the MYBPC3 regulatory domain. Critical sites in MYBPC3 are hinges (one known another proposed) sterically enabling regulatory interactions with βmys. The critical site in βmys is the actin binding C-loop (residues 359-377), a contact sensor triggering actin-activated myosin ATPase and contraction velocity modulator coordinating with actin bound tropomyosin [22]. C-loop and MYBPC3 regulatory domain linkage potentially impacts multiple functions across the contractile system. Co-domain identification in a multiprotein complex maps path-of-influence mechanical coupling across the βmys/MYBPC3 interface [23] and implies potential to use them for native in vivo proximity constraints.

## Results

### Co-domain interaction by real or virtual mechanisms

**Fig. 3:**
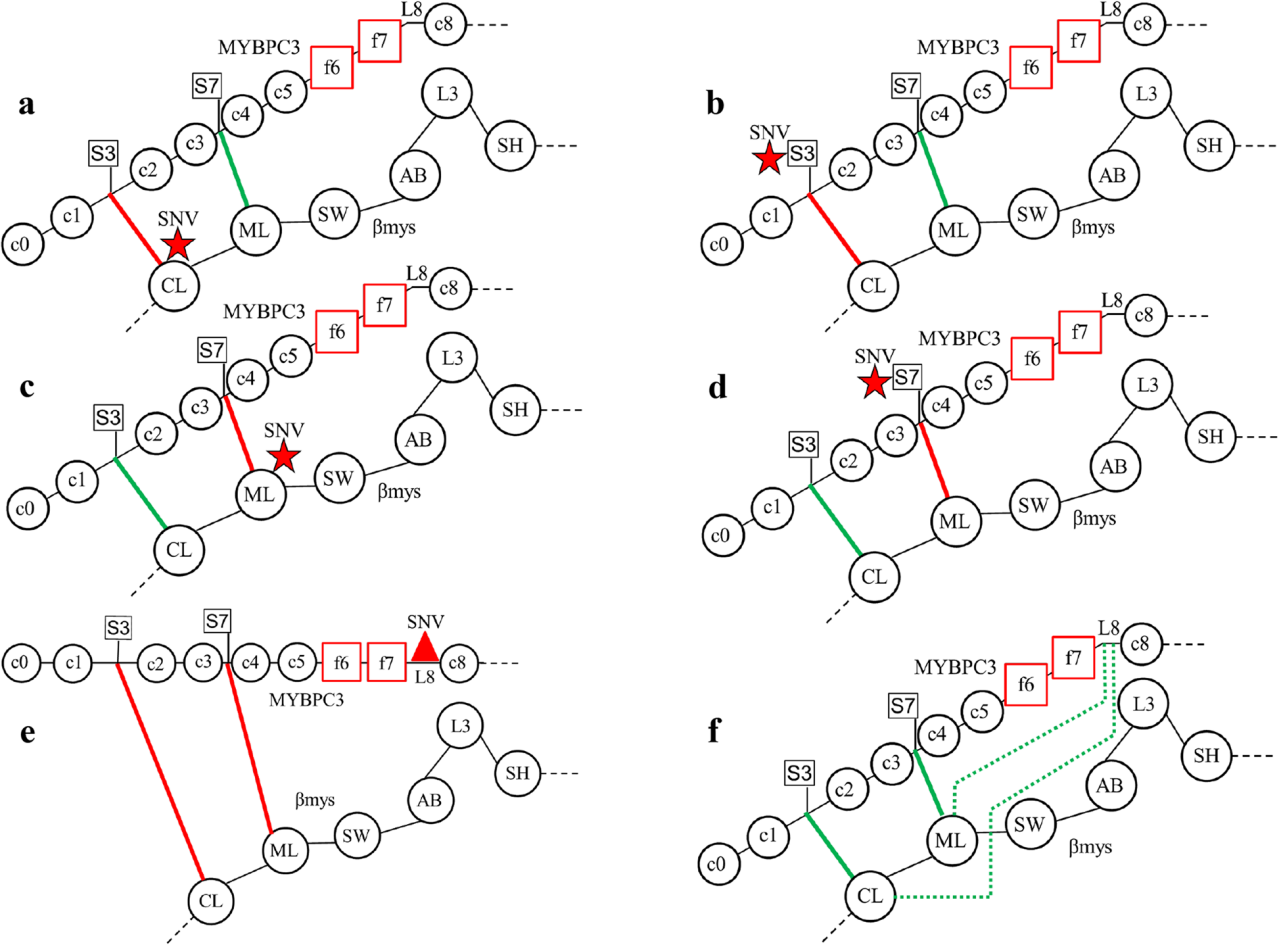
Hypothetical βmys/MYBPC3 complexes in human populations for SNVs detecting real and virtual co-domains. Functional domains CL and ML in βmys, and, functional domains S3 and S7 in MYBPC3 are SNV modified individually as shown in **a**, **c**, **b**, and **d**. Complexes SNV (red star) modified at C-loop (**a**), phosphorylatable serine S3 (**b**), myopathy loop (**c**), or phosphorylatable serine S7 (**d**) are species needed in the database to surmise the co-domain interactions in the native species shown in **f**. The native complex involves real co-domains indicated by the solid green lines between S3 and CL and between S7 and ML. Co-domain interruption by SNVs is indicated by the solid red lines connecting S3-CL or S7-ML in **a-d**. SNV modification in **e** locates to L8 (red triangle) where it alters MYBPC3 conformation to interrupt real co-domains S3-CL and S7-ML. The virtual co-domains L8-CL and L8-ML are indicated by the broken green lines in **f**

Any co-domain pair from two proteins, A and B, in complex implies two functional domains (one in A and one in B) where SNV modification probabilities cross-correlate over human populations. It occurs by two mechanisms named real and virtual. A hypothetical example (Fig. 3) demonstrates the real mechanism in a complex of proteins A and B where A is βmys and B MYBPC3. Consider functional domains CL and ML in βmys, and, S3 and S7 in MYBPC3. They are SNV modified individually as shown in Fig. 3a-d and disrupt the real S3-CL or S7-ML co-domain interactions. This is indicated by a solid red line connecting the domains. Detecting real co-domain combinations S3-CL and S7-ML requires detecting four SNVs at S3, S7, CL and ML. Favorable cross-correlation of their SNV modification probabilities implies real co-domain interaction indicated by solid green lines in Fig. 3f.

The virtual mechanism has a SNV altered functional domain in protein B that perturbs one or more real co-domain contacts between proteins A and B in complex. Real co-domain contacts do not involve the SNV altered functional domain in protein B. This scenario is also depicted in Fig. 3. Consider the functional domains CL and ML in βmys, and, L8 in MYBPC3. They are SNV modified individually as shown in Fig. 3a, c, and e. The SNV modification of L8 alters MYBPC3 conformation to disrupt real co-domains S3-CL and S7-ML. This is indicated by solid red lines connecting the domains (Fig. 3e). Detecting virtual co-domains combinations L8-CL and L8-ML involves detecting three SNVs at L8, CL, and ML. Favorable cross-correlation of their SNV modification probabilities implies virtual co-domains L8-CL and L8-ML indicated by the broken green lines (Fig. 3f).

Real vs virtual mechanisms for co-domain interaction identified by SNV probability cross-correlation are indistinguishable without additional information. Real co-domains involve a pair of cross-protein domains while virtual co-domains involve multiple domain interactions. Real co-domains coordinate across complexed proteins requiring a binary cooperation that would confer more specificity. Virtual co-domains sometimes involve multiple interactions.

### Complex βmys/MYBPC3

**Fig. 4:**
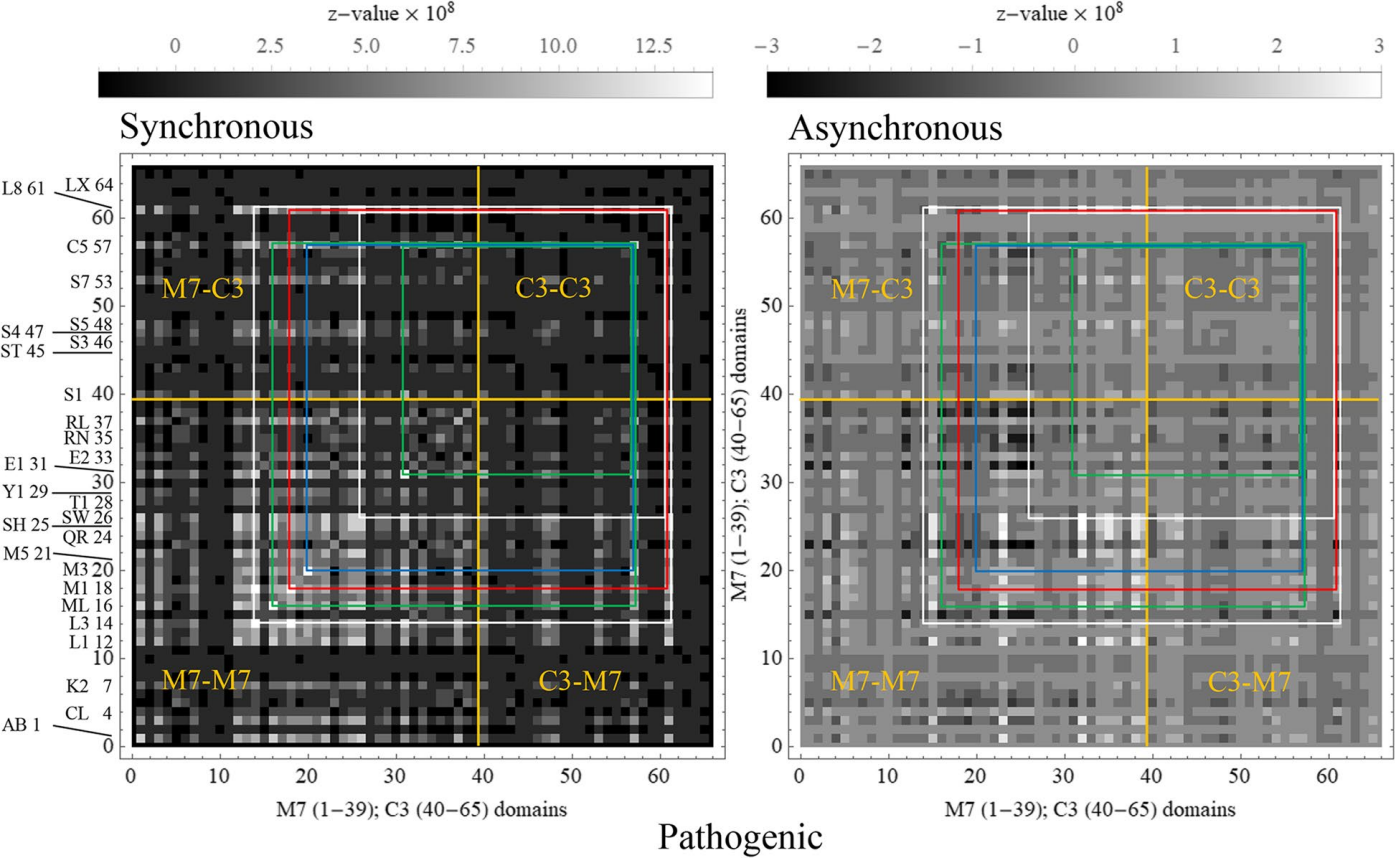
βmys/MYBPC3 complex correlation maps for pathogenic outcomes implied by 2D-CG. Synchronous (left) or asynchronous (right) maps have axes identical for both dimensions representing βmys (M7 1-39) followed by MYBPC3 (C3 40-65) domains. Domain index is linked to its two-letter code and protein sequence in Supplementary Table S1. Two-letter codes for domains label some indices on the leftmost axis in the figure. Intensities (z-values) are indicated numerically by the grayscale. Regions defined by vertical and horizontal orange lines at the interface of pixels 39-40 separate intra-protein cross-peaks (within regions M7-M7 and C3-C3) from inter-protein cross-peaks (within regions M7-C3 and C3-M7). Intensity peaks along the diagonal in the synchronous map are autocorrelated probabilities for each domain. Correlation squares link the 6 most significant off-diagonal co-domain coordinates falling within the M7-C3 and C3-M7 regions. They are white, green, red, blue, for different βmys domains then repeating color sequence as needed

Figure 4 shows 2D-CG maps for complex βmys/MYBPC3 with pathogenic outcomes. Each pixel corresponds to one co-domain pair. The 2D-CG synchronous intensity map (left) identifies domain pairs whose SNV probabilities correlate or anti-correlate for identical populations while the 2D asynchronous intensity map (right) identifies the leading and lagging co-domain member over populations ordered by decreasing genetic divergence. The 6 most significant pathogenic co-domain interactions have combined synchronous and asynchronous co-domain cross-correlates that are >5.8 standard deviations from the mean. Correlation squares linking these co-domain fall within the M7-C3 and C3-M7 regions related by inversion through the diagonal line.

**Fig. 5:**
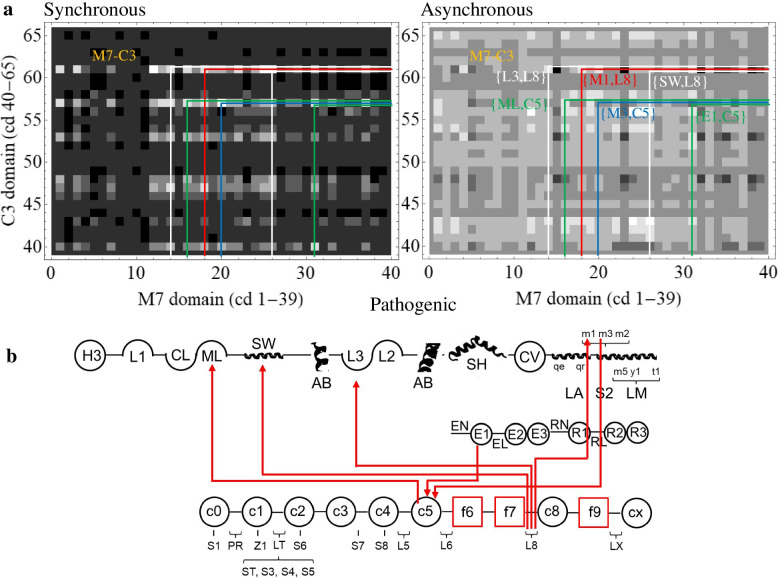
βmys/MYBPC3 complex cross-correlation maps and their implied inter-protein domain linkages for pathogenic outcomes. **a** M7-C3 region for synchronous and asynchronous maps and the portion of the correlation squares falling within them for pathogenic outcomes taken from Fig. 4. Co-domain ordered pairs corresponding to each correlation square are indicated in parenthesis for M7 domain and C3 domain in first and second positions, respectively. Ordered pairs are color coded to match the corresponding correlation square. **b** Red lines show most significant directed co-domain interactions between βmys and MYBPC3 for pathogenic outcomes. Nomenclature otherwise identical to Fig. 4

Figure 5a zooms in on the M7-C3 region for synchronous and asynchronous maps from Figs. 4 and 5b red lines represent co-domain interactions linking βmys and MYBPC3 for both synchronous and asynchronous correlates with the arrow indicating asynchronous pathway phase where leading or lagging (lagging at the pointy end) is relative to the human population sequence from Supplementary Table S7. Synchronous pathways are also directional but represent simultaneously increasing SNV probabilities in the co-domains for all cases in this study. Pathways identify inter-protein transduction interrupted by pathogenic SNVs. Highest significance synchronous and asynchronous pathways involve MYBPC3 linker 8 (L8) or Ig-like domain 5 (C5) and six diverse βmys domains. L8 leads asynchronous interactions with βmys switch 2 helix (SW), actin binding loop 3 (L3), and the βmys-S2 binding site for MYBPC3-C1 (M1) [24]. C5 leads asynchronous interaction with βmys myopathy loop (ML), and, lags asynchronous interactions with ELC EF1 domain (E1) and the βmys-S2 binding site for MYBPC3-C2 (M3) [25]. SNVs contributing to the map are pathogenic and affect either the M7 or C3 side at the ends of the red lines.

The L8 domain in MYBPC3 has co-dependence with multiple sites on βmys suggesting the virtual-mechanism wherein a SNV altering the co-domain in MYBPC3 perturbs multiple inter-protein contacts with βmys. Hypothetically L8 functions as a hinge in MYBPC3 while in close proximity to βmys-LM while the C8-CX domains anchor MYBPC3 to the thick filament [15]. Similar logic applies to C5 where its co-dependence with multiple sites on βmys likewise suggests the virtual-mechanism. The C5 domain in MYBPC3 was already identified as at or near to a hinge point [16] supporting the virtual co-domain hypothesis.

**Fig. 6.**
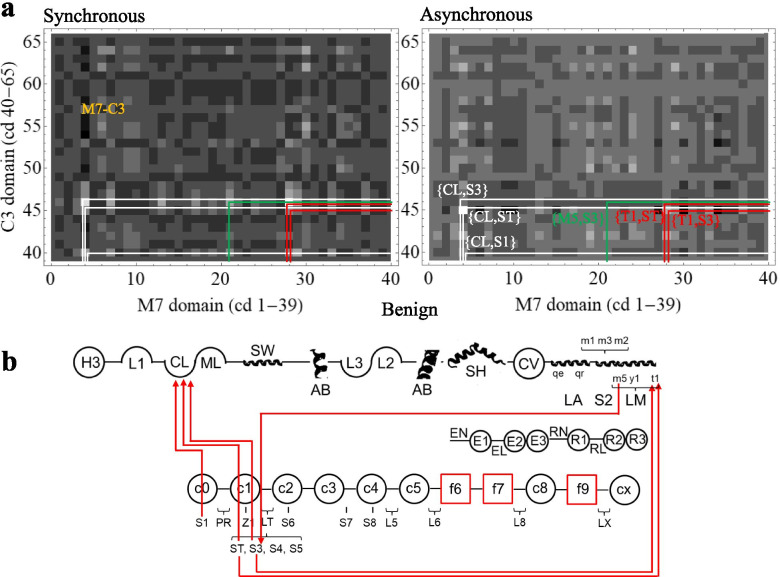
βmys/MYBPC3 complex cross-correlation maps and their implied inter-protein domain linkages for benign outcomes. **a** M7-C3 region for synchronous and asynchronous maps and the portion of the correlation squares falling within them for benign outcomes taken from Supplementary Figure S4. **b** Red lines show most significant directed co-domain interactions between βmys and MYBPC3 for benign outcomes. Nomenclature otherwise identical to Figs. 4 and 5

Figure 6 parallels Fig. 5 but for benign outcomes. The 6 most significant co-domain interactions have combined synchronous and asynchronous co-domain cross-correlates that are >3.7 standard deviations from the mean. Figure 6b red lines show co-domain interactions linking βmys and MYBPC3. Highest significance synchronous and asynchronous pathways involve MYBPC3 phosphorylatable serines S1(Ser47), ST(Ser273), and S3 (Ser282) and three domains of βmys. ST and S3 in the MYBPC3 regulatory domain and S1 lead asynchronous interactions with βmys actin binding C-loop (CL). ST and S3 lead asynchronous interactions with βmys-LM titin binding site (T1) [26, 27]. S3 lags an asynchronous interaction with βmys-LM MYBPC3-CX binding site (M5) [26]. SNVs contributing to the 2D map are benign and affect either the M7 or the C3 side at the ends of the red lines.

The actin binding C-loop domain in βmys regulates actin-activated myosin ATPase [28] and modulates contraction velocity in coordination with actin bound tropomyosin [22]. It engages the MYBPC3 regulatory domain in linker 2 (LT) and S1 in C0 suggesting joint βmys/MYBPC3 regulation of actin-activated myosin ATPase and actomyosin translation speed. Other co-domains identified involve MYBPC3 sites in LT interacting with βmys sites in LM mostly near the βmys C-terminus recalling the transient binding of the MYBPC3 N-terminal peptide C0-C1-LT with the thick filament [29].

### Control complex MAPT/MYBPC3

**Fig. 7.**
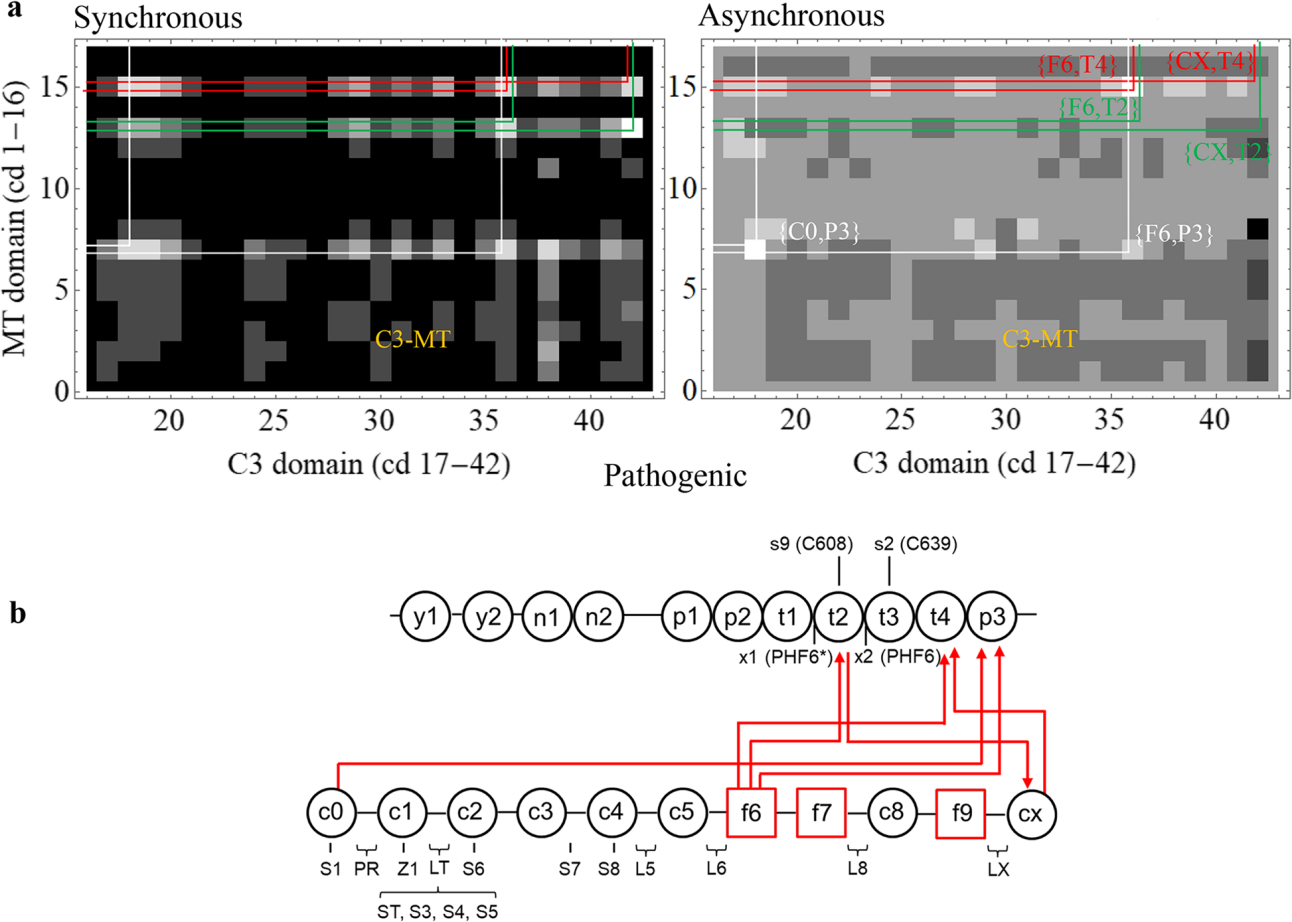
MAPT/MYBPC2 complex cross-correlation maps and their implied inter-protein domain linkages for pathogenic outcomes. **a** C3-MT region for synchronous and asynchronous maps and the portion of the correlation squares falling within them for pathogenic outcomes taken from Supplementary Figure S5. **b** Red lines show most significant directed co-domain interactions between MAPT and MYBPC3 for pathogenic outcomes. Nomenclature otherwise identical to Figs. 4 and 5

**Fig. 8.**
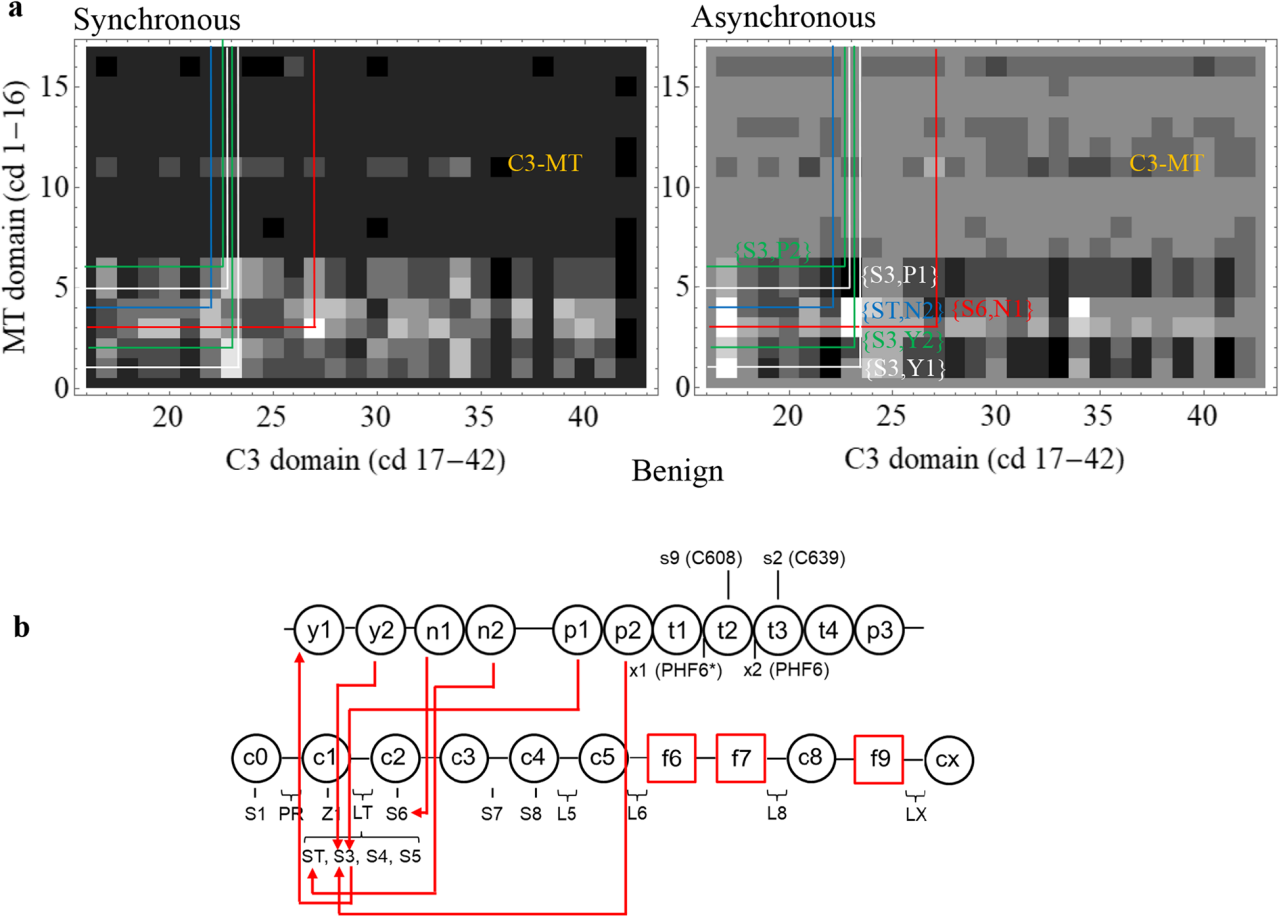
MAPT/MYBPC2 complex cross-correlation maps and their implied inter-protein domain linkages for benign outcomes. **a** C3-MT region for synchronous and asynchronous maps and the portion of the correlation squares falling within them for benign outcomes taken from Supplementary Figure S6. **b** Red lines show most significant directed co-domain interactions between MAPT and MYBPC3 for benign outcomes. Nomenclature is otherwise identical to Figs. 4 and 5

A control computation on a system that presumably never forms a natural functional entity, complex MAPT/MYBPC3, is identical to that done for complexed βmys/MYBPC3. Microtubule-associated protein tau (MAPT) is an intrinsically disordered protein regulating microtubule formation from tubulin [30]. Tau amyloid aggregation associates with tau hyperphosphorylation [31] and neurodegenerative diseases including Alzheimer’s [32]. Alternative splicing variants of the MAPT gene express eight isoforms of the protein (isoforms 1-8) that have SNVs in the National Center for Bioinformatics (NCBI) SNP database. MAPT structural organization for the 758 kDa molecular mass variant (isoform 1) is shown in Fig. 2. Other isoforms are shorter principally by deletion of t2 with 2, 1, or 0 N-terminal inserts. Tau aggregation beginning with paired helical filament (PHF) formation involves cysteins C608 and C639 [33] and hexapeptide motifs PHF6* and PHF6 [34]. MAPT and MYBPC3 collectively have 42 domains, 36 population classifications, and 30 phenotype classifications listed in Supplementary Table S4, Table S5, and Table S6. Figures 7a and 8a show the C3-MT regions for the synchronous and asynchronous maps. The 6 most significant co-domain cross-peaks for pathogenic and benign outcomes are >4.1 and >2.3 standard deviations from their mean. These data are summarized by the co-domain interactions (Figs. 7b and 8b) indicating potential inter-protein transduction pathways infiltrated by pathogenic or benign SNVs.

Significance testing for results (Figs. 5, 6, 7 and 8) is described in Methods. It measures βmys/MYBPC3 co-domain correlates statistical significance relative to control. The relative *p*-values defined there are 0.003 and 0.03 for pathogenic and benign cases. Values ≤0.05 are significant implying co-domain correlates from βmys/MYBPC3 are significantly different from control.

## Discussion

Cardiac muscle sarcomere proteins comprise the molecular machinery generating and regulating muscle contraction. Their coordinated action involves most critically the βmys motor powering contraction by ATP free energy transduction into mechanical work and MYBPC3 in a phosphorylation dependent regulatory role. This contractile system subset dynamically adapts contractile force and velocity using myosin step-size modulation [1, 35] and a coordinated βmys/MYBPC3 interaction [11]. Familial hypertrophic cardiomyopathy is most often associated with mutation in one of these two proteins affirming their significance in cardiac muscle contraction [36].

Structural information pertaining to adaptive contractile force and velocity is often static and at highest resolution when individual sarcomere proteins are crystallized [37] or at usually lower resolution when larger reconstituted systems are frozen for cryoEM [38]. 2D-NMR structures include dynamic characteristics by using structural ensembles generated with constrained molecular dynamics simulation [39]. In vivo approaches include single zebrafish skeletal myosin motor dynamics in isometric contraction [40] and βmys motor step-size dynamics in the beating zebrafish heart [35]. The computational approach introduced here involves worldwide genetics data from human hearts in vivo where SNV residue substitutions probe βmys or MYBPC3. The NCBI SNP database contains results from this large-scale natural experiment.

βmys/MYBPC3 and MAPT/MYBPC3 interactions were investigated in this study. The former is the known complex in a working heart. The latter is unlikely to form in vivo hence its relevance as a control. Inter-protein domains correlated by human population in 2D identify co-domains in the βmys/MYBPC3 complex as shown in Figs. 4, 5 and 6 for pathogenic and benign SNVs. Pathogenic SNVs alter gene product sequence impairing functional pathways through co-domains. The pathogenic case implies co-domains lose functional integrity when modified over the shifting genetic context of human populations. Benign SNVs alter gene product sequence without impairing functional pathways through co-domains. The benign case implies co-domains maintain functional integrity when modified even over the shifting genetic context of human populations. The 6 highest significance co-domains for the complex for either pathogenic or benign pathogenicities are identified as the cross-peaks in correlation squares for synchronous and asynchronous interactions. Synchronous/asynchronous nomenclature refers to genetic divergence ordered human populations. It identifies domain pairs with SNV probability of one member synchronize with, leading, or lagging the other member over populations ordered by quantitation of their genetic divergence. Leading domain SNV probabilities are more affected by populations containing higher genetic divergence vs the lagging domain where SNV probabilities are more impacted by populations containing lower genetic divergence.

Cross-peaks linking βmys with MYBPC3 indicates co-domain coupling (Fig. 5a). Co-domain maps (Fig. 5b) interpret these data for combined synchronous and asynchronous interactions of pathogenic SNVs. It shows 3 directed pathways linking L8 in MYBPC3 with actin binding (L3), energy transduction (SW), and C1 binding (M1) [14] related domains in βmys. The peptide linker in the MYBPC3 N-terminus between C1 and C2 (LT) has phosphorylation sites participating in contractile regulation by modulating myosin activity [9–11] and calcium sensitivity [12]. It is reasonable to assume that disrupting C1 binding to βmys could impact the regulatory function on contraction exerted by LT. The L8-βmys interaction is the model for the virtual co-domain mechanism where a SNV altering structure in MYBPC3 L8 affects coupling to multiple real co-domains in βmys (Fig. 3).

Three directed pathways link SNVs in C5 from MYBPC3 with actin binding (ML), force generation (E1) [6, 41], and C2 binding (M3) [14] related functional domains in βmys (Fig. 5b). Like with L8, it is interpreted as indicating SNVs in C5 disrupt multiple co-domain interactions involving ML, E1, and regulatory domain LT. It again implies the virtual co-domain effect but involving C5 wherein a SNV altering structure in MYBPC3 C5 affects coupling to multiple real co-domains in βmys.

MYBPC3 LT and hinge points near C5 were identified independently corroborating the hypothesis that SNVs associated with C5 can disrupt bending in the MYBPC3 that is key to forming real co-domain interactions in complex βmys/MYBPC3 [16]. A similar role proposed here for linker 8 (L8) implies it is a third flexible linker. Figure 6 from Lee et al. (2015) [29] compared with Fig. 4 from Previs et al. (2016) [16] implies the L8 hinge but the implication is not interpreted by the authors. Nonetheless, it is improbable that MYBPC3 functions without a hinge at or near L8 to allow the multiple co-domain interactions proposed for the βmys/MYBPC3 complex while the MYBPC3 C-terminus is bound to LM on the thick filament.

Cross-peaks linking βmys with MYBPC3 indicate co-domain coupling (Fig. 6a). Co-domain maps (Fig. 6b) interpret these data for combined synchronous and asynchronous interactions of benign SNVs. The maps show directed pathways link SNVs in βmys at the C-loop (CL) actin binding site to regulatory phosphorylation sites in the regulatory domain of MYBPC3 at LT and in C1. It implies and confirms the regulatory control mechanism at LT in MYBPC3. It also implies participation of the C-loop in βmys/MYBPC3 modulated force/velocity regulation. The C-loop participates in energy transduction [28], actin-activation of myosin ATPase [42] and modulation of myosin in vitro motility velocity in the presence of actin bound tropomyosin [22]. The latter was confirmed in a static structure of a thick filament [43]. The C-loop maintains resilient links with the MYBPC3 regulatory domain.

Directed pathways involve SNVs in βmys at LM and in MYBPC3 at regulatory domain LT (Fig. 6b). LM sites at M5 and T1 engage with ST, S3, or both. M5 and T1 are binding sites for CX (MYBPC3) and titin [26, 27], respectively. It implies that several sites on LM maintain interactions with S3, a principal phosphorylatable serine in the MYBPC3 regulatory domain. The βmys C-loop and LM maintain robust lines of communication with the regulatory phosphorylation sites in the MYBPC3 regulatory domain at LT and in C1 that stand despite SNV modification and under changing genetic background of human populations.

The evidence suggests pathogenic vs benign co-domains selectively identify mechanical vs regulatory transduction functions within the complex. Co-domains in βmys associated with its mechanical function are less resilient to SNV modification and become less reliable with decreasing genetic divergence, i.e., migration out of Africa.

The analysis described for βmys/MYBPC3 was applied to the control complex MAPT/MYBPC3. These data are summarized in Figs. 7 and 8. Highest pathogenic and benign thresholds for 0 co-domains in complex MAPT/MYBPC2 are 4.7 and 2.7 standard deviations above mean compared to lowest thresholds for 6 co-domains in βmys/MYBPC3 of 5.8 or 3.7 standard deviations above mean. Contrasting thresholds imply there are no false positives for co-domains in complex βmys/MYBPC3. Minima of 1.1 and 1.0 standard deviations separate a system with known inter-protein contacts from one with (presumably) none suggesting the headroom distinguishing them. Methods describes relative significance tests that compare βmys/MYBPC3 and control complexes giving relative p-values of 0.003 or 0.03 for pathogenic or benign SNVs. Both are ≤ 0.03 implying βmys/MYBPC3 cross-correlates differ from control with ≥97% certainty.

Opportunity for insight into protein complex structure/function using 2D-CG rests on the accuracy of earlier work and the SNV database. The approach mines genetic data and interprets it in a hypothesis driven model. The model involves three features in the data set: (i) variant locations in co-domains, (ii) variant population group, and (iii) hypothetical variant probability perturbation by genetic divergence over population groups. The hypothetical perturbation correlates functional associations between interacting protein co-domains in the ventricular cardiac sarcomere. Ethical standards limit in vivo experimentation on humans. It confines experimental data to that from the NCBI SNV database or equivalent sources. This data is collected worldwide implying uniformity and reliability vary. That different population groups have diverse genetic makeup is evident, however, whether population groups can be ordered in a linear variation of genetic divergence to act as the perturbation in 2D-CG is an assertion supported by earlier work [19, 44, 45]. Similarly, the model hypothesis asserts that genetic divergence imposes diverse genetic backgrounds impacting cardiac muscle physiology in the form of functional associations between interacting proteins sub-domains is likewise supported by earlier work [17, 18]. Finally, the linear relationship between genetic divergence and population group is quantitative and repeatable but also depends on data like that from the NCBI SNV database. These considerations imply potential sources for systematic error, however, parallel investigation of the control system circumscribes random uncertainty by statistical accuracy. Limitations indicated here apply to most or all human research that involves in vivo conditions.

Future application of 2D-CG might involve other sarcomere proteins such as human cardiac tropomyosin (TPM1), troponin T (TNNT2), troponin C (TNNC1), troponin I (TNNI3), and actin (ACTC1) containing 466, 313, 154, 399, and 210 missense SNVs compared with 2001 and 1436 for MYH7 and MYBPC3 (comparing SNV counts from the current NCBI SNP database build). Actin and actin associated regulatory proteins are promising candidates for study.

## Conclusion

SNVs change protein sequences in βmys and MYBPC3 modifying protein domain structure and function. These are the physical cause of inheritable heart disease. When a SNV modified domain locates to the point of contact in the βmys/MYBPC3 complex it modifies the co-domain interaction purposed to coordinate function. The bilateral nature of a co-domain implies a joint impact from modification at one end or the other suggesting that a joint statistical analysis investigating correlated responses to a common perturbation should identify their presence. Genetic divergence over human populations is the perturbation causing the SNV probability coupling in this method called 2D-CG. Pathogenic and benign SNV data implies three co-domain hubs, C5 and L8, in MYBPC3 with links to several domains across the βmys motor, and, C-loop in βmys with links to the MYBPC3 regulatory domain LT. C5 links with actin binding, force generation, and C0-C2 binding sites in βmys. L8 links with actin binding, energy transduction, and C0-C2 binding sites in βmys. These critical sites in MYBPC3 are known (C5) and proposed (L8) regions that bend. The critical site in βmys (C-loop) is an actin binding site, an actin contact sensor regulating actin-activated myosin ATPase, and a contraction velocity modulator related to its interaction with actin bound tropomyosin. Links between C-loop and LT impact the principal functions of the cardiac contractile system. The identification of co-domains in a multiprotein complex implies a potential to estimate spatial proximity constraints for dynamic protein interactions in vivo.

## Methods

### SNV data retrieval and analysis

Supplementary Figure S1 outlines the protocol for data retrieval from NCBI. Summary descriptions of the analytical steps leading to the 2D-correlation maps (Figs. 4, 5, 6, 7 and 8) are in sections that follow.

### Neural/Bayes network configuration

A directed acyclic graph (DAG) (Supplementary Figure S2a) describes the trial network configurations associating mutant location in the protein domain, residue substitution, population group, and SNV allele frequency in a causal relationship with phenotype and pathogenicity as described previously [17].

Protein domains and their 2 letter abbreviations are indicated in Supplementary Table S1. A protein complex made from four genes, MYH7, MYL2, MYL3, and MYBPC3 has domains from 65 functional sites combining assignments made previously [46] and new assignments in myosin including blocked head/converter interface (BH, index 3) [47], mesa trail (MR, index 17) [48], binding sites for C1, C2, and LT in MYBPC3 on myosin S2 (M1, M3, and M2, indices 18, 20, and 19) [24, 25], binding site for CX in MYBPC3 on myosin LM (M5, index 21) [26], binding sites for titin and myomesin on myosin LM (T1 and Y1, indices 28 and 29) [26, 27, 49], and for subdomains in RLC (RN, R1, RL, R2, and R3, indices 35-39) [50]. Every SNV in the database has an assigned domain. Figure 2 shows linear representations of βmys and MYBPC3 indicating mutual binding sites and the locations of most domains listed in Supplementary Table S1.

Residue substitution refers to the SNV reference and substituted residue (ref/sub) pair. Ref/sub combinations have 420 possibilities for 21 amino acids. Pooled categories related to size, hydrophilicity, and charge such that (Arg, His)→Lys, Asp →Glu, Ser →Thr, Asn →Gln, (Ala, Val, Ile, Met, Phe, Trp) →Leu reduce combinations to 59.

Human population group and allele frequency fill out the independent parameters in the network. Supplementary Table S2 indicate populations and their 3 letter abbreviations. Allele frequency (af) is a continuous variable in the database on the interval 0 ≤ af ≤ 1 for 1 meaning all alleles are substituted by the SNV. These data are divided into two discrete categories of ≤1% (category 0) or >1% (category 1).

The NCBI SNP database has 36 phenotype data classifications for cardiovascular disease pertaining to βmys and MYBPC3 variants. Classifications change over time with additions and subtractions. Supplementary Table S3 lists names and two letter codes. Most phenotypes associate with both βmys and MYBPC3 SNVs. Data submissions from different providers occasionally conflict for a given SNV. Pathogenicity data classifications include pathogenic (pt), likely pathogenic (lp), benign (be), likely benign (lb), and unknown (uk). Phenotype and pathogenicity category assignments from different providers sometimes conflict. They are assigned from the pool when there is a clearly dominant choice. In all other cases the unknown category is assigned.

### Neural network validation

The neural network indicated in Supplementary Figure S2b and S2c models structure/function influences from disease and follows from the DAG in Figure S2a and as described previously [17]. The NCBI SNP database was mined to assign the known independent and dependent discrete variables for βmys and MYBPC3 in 6-dimensional data points (fulfilled 6ddps). They train and validate the neural network. The majority of 6ddps have one or both dependent data points unknown (unfulfilled 6ddps). The neural network model predicts unknowns. Independent discrete physical variables are always knowns in the 6ddps.

Training and validating neural network models of contraction were described [17]. It provides 20 best-of-the-best models that are distinct implicit models for the role of complex βmys/MYBPC3 in transduction. The selection process is suitable for minimizing random error but is unlikely to address systematic model limitations. Models exactly reproduce ≥60% of the known 6ddps in the target protein complex constraining potential systematic errors in the models to <40% of the dataset and implying the measure of their accuracy. Systematic error increased substantially together with phenotype category expansion. The difficulty is due to the wide variety of assigned phenotypes while just a very few predominate (e.g., familial hypertrophic cardiomyopathy and cardiomyopathy). Enlarging the independent parameter set, for instance by enlarging the different residue substitutions to 420 (vs current 59) possibilities for 21 amino acids, and expanding depth and connectivity in the neural network models are likely to reduce error and will be addressed in future work.

### Bayes network modeling of complexed βmys/MYBPC3 transduction mechanism

Supplementary Figure S2a shows the DAG for the Neural/Bayes network model. Arrows imply a direction for influence hence the domain, residue substitution, population, and allele frequency assignment collectively imply probability for phenotype and pathogenicity. Datasets 6ddpbmysMYBPC3.xls and 6ddpMAPTMYBPC3.xls in Supplementary Data contain fulfilled and unfulfilled 6ddps for complexed βmys/MYBPC3 and MAPT/MYBPC3 with 39,290 and 31,181 total variations, respectively. Combined fulfilled and predicted 6ddp data sets define conditional probabilities for the systems in the form of conditional probability tables (CPTs). The product of conditional probabilities on the right defines the joint probability density on the left in,

1 $$P\left[ cd,\kern0.5em re\kern0.5em , po,\kern0.5em af,\kern0.5em ph,\kern0.5em pa\right]\equiv P\left( pa\left| ph,\kern0.5em cd,\kern0.5em re,\kern0.5em po,\kern0.5em af\right.\right)P\left( ph\left| cd,\kern0.5em re,\kern0.5em po,\kern0.5em af\right.\right)$$

Calculating SNV probability for domain *i* in population *j* uses joint probability density,

2 $$p\left\{{cd}_i,{po}_j,{pa}_k\right\}=\sum_{af, re, ph}P\left({pa}_k| ph,{cd}_i, re,{po}_j, af\right)P\left( ph|{cd}_i, re,{po}_j, af\right)$$

where summation is over all values for allele frequency, residue substitution, and phenotype.

### 2D correlation testing and significance

SNVs from human population *po*_*j*_ reside in a protein domain *cd*_*i*_ with probability given by Eq. 2. Furthermore, pathogenicity is made binary, either pathogenic or benign, by combining likely-pathogenic with pathogenic probabilities or likely-benign with benign probabilities (Eq. 2 and k=lp, pt or be, lb respectively). In this scenario, Eqs. 2 specializes into,

3 $$p\left\{{cd}_i,{po}_j, pathogenic\right\}=\sum_{af, re, ph,k= lp, pt}P\left({pa}_k| ph,{cd}_i, re,{po}_j, af\right)P\left( ph|{cd}_i, re,{po}_j, af\right)$$

4 $$p\left\{{cd}_i,{po}_j, be nign\right\}=\sum_{af, re, ph,k= lb, be}P\left({pa}_k| ph,{cd}_i, re,{po}_j, af\right)P\left( ph|{cd}_i, re,{po}_j, af\right)$$

Equations 3 and 4 expressions populate the synchronous and asynchronous generalized 2D correlation intensities [20],

5 $$\Phi \left\{{cd}_i,{cd}_j, pa\right\}=\frac{1}{np-1}\sum_kp\left\{{cd}_i,{po}_k, pa\right\}\ p\left\{{cd}_j,{po}_k, pa\right\}$$

6 $$\Psi \left\{{cd}_i,{cd}_j, pa\right\}=\frac{1}{np-1}\sum_{k_1}p\left\{{cd}_i,{po}_{k_1}, pa\right\}\kern0.5em \sum_{k_2} nf\left[{k}_1,{k}_2\right]\kern0.5em p\left\{{cd}_j,{po}_{k_2}, pa\right\}$$

for,

7 $$nf\left[{k}_i,{k}_j\right]=\left\{\begin{array}{c}0,\kern3.75em {k}_i={k}_j\\ {}\frac{1}{\pi \left({k}_j-{k}_i\right)}, otherwise\end{array}\right.$$

Φ in Eq. 5 gives synchronous, and Ψ in Eq. 6 asynchronous, 2D correlation intensities for *np* the number of populations represented by *po*, and *pa* either pathogenic or benign. The 2D synchronous intensity map will identify domain pairs whose SNV probabilities correlate or anti-correlate for identical populations while the 2D asynchronous intensity map identifies the leading and lagging co-domain member over populations ordered by their genetic divergence.

Data obtained from calculation of protein domain SNV probability correlations is represented in 2D plots showing Φ or Ψ for the identical listing of first βmys (abbreviated M7) then MYBPC3 (C3) domains, in the order given in Supplementary Table S1 for a total of 65 domains, on both x- and y-axes. Domains are discrete entities hence the 2D plots resemble a pixelated image with grayscale representing intensity. Both intra- and inter-protein correlations are indicated with domain autocorrelated intensity on the diagonal.

The most significant combined synchronous and asynchronous co-domain interaction cross-correlates are the largest elements in the array,

8 $${a}_{i,j}=\left|\Phi \left\{{cd}_i,{cd}_j, pa\right\}\right|+\pi\ \left|\Psi \left\{{cd}_i,{cd}_j, pa\right\}\right|$$

for Φ and Ψ from eqs. 5 and 6, *cd*_*i*_ and *cd*_*j*_ the SNV containing functional domains in βmys and MYBPC3, and *pa* their common pathogenicity (pathogenic or benign). Array element *a*_*i,j*_ is the co-domain cross-correlate amplitude given by the product of probabilities for SNVs in each domain member of the co-domain weighted by synchronous or asynchronous coupling to human populations. Quantity π multiplying the asynchronous cross-correlates (Ψ) in Eq. 8 balances weighting for synchronous (Φ) with nearest neighbor population asynchronous cross-correlates. Elements *a*_*i,*j_ are ≥ 0. They are combined with *-a*_*i,j*_ then their distribution visualized in a histogram. A normal distribution approximates the result. Distance from the mean expressed in multiples of standard deviation indicates a measure of co-domain cross-correlate amplitude significance. Co-domain cross-correlate amplitude significance increases with distance from amplitude mean.

Comparing results from MAPT/MYBPC3 (control) and βmys/MYBPC3 (unknown) complexes estimates significance. Co-domain cross-correlate amplitude distribution for the control has an area under the positive tail of the distribution curve for hypothetical cross-correlates just exceeding the most significant observed co-domain cross-correlate amplitude. It estimates probability for amplitudes lying beyond controls where unknown actual cross-correlates reside. Next, the same method estimates area under the positive tail for the least significant co-domain cross-correlate amplitude within the 6 most significant βmys/MYBPC3 co-domain cross-correlate amplitudes. The least significant βmys/MYBPC3 amplitude differs significantly from control provided area for the unknown divided by the control area, called relative *p*-value, is ≤0.05.

Population dependence for the most significant co-domain interactions in βmys/MYBPC3 apparently excludes 4 population groups (PCC, EUA, SAS, and AFM, Supplementary Figure S7). Its significance needs further study.

### Population genetic divergence proxy

Worldwide human genetic divergence attributed to migration from a single origin in East Africa is based on the serial founder effect addressing migration [19], colonization, and exchange between geographically near populations [45]. The serial founder effect explains the observed linear divergence decrease with human migration distance over the earth’s surface. Linearly decreasing genetic variation consistent with the serial founder effect is likewise detected using a SNV database independently characterizing worldwide genetic variation [44]. Migration distance is therefore a good proxy for genetic divergence variation.

Estimates for migration distances on earth’s surface use two methods. The first uses great circle distances between waypoints following pathways described by Ramachandran et al. (2005) [19] but with some waypoint additions. The second uses driving distances between the same waypoints where feasible. The former is shorter than the latter without exception. Together they estimate lower and upper bounds to migration distance that are adjusted incrementally outward from their midpoint until a line fits through population migration distances within the new bounds. Upper and lower bounds are factors of 1.12 and 1.14 larger than their initial estimates for populations listed in Supplementary Tables S7 and S8. The latter are subsets of the human populations in Supplementary Tables S2 and S5 made by eliminating those for which distance ranking is not feasible (GLO, OTH) or migration distance redundant (TWC & PCC are both from people in the UK and only PCC was included because it is the larger dataset). Distance estimates (and genetic divergence variation) from the fitted line for populations in Supplementary Tables S7 and S8 are equally spaced over the Population Index parameter as needed for the co-domain 2D-CG formalism.

Supplementary Figure S3 summarizes migration pathways starting from Addis Ababa, Ethiopia and ending at points used to estimate migration distance for populations. Migration to all destinations are via Cairo, Egypt except those ending in Africa. Europe is reached from Cairo by two pathways, one via Istanbul, Turkey then west, and, second from Asia after a northerly detour east of the Caspian Sea via Chelyabinsk, Russia. Three routes reach East Asia, two north and one south of the Himalayan mountains. A fourth Asian pathway through northern Russia, and more recent forced or voluntary immigration from Africa, Europe, and China, reaches North and South America. The south Asian pathway and immigration from England reaches Oceania.

Individuals sampled in North America, South America, and Australia contributed SNV data to the NCBI database from native and non-native populations. Native and non-native path distance with weights proportional to the ethnic contributions to local population estimated overall migration distance to these regions. Two or more race (mixed) individuals contribute fractional parts to each represented single race population (Asian, Black Africans, Caucasian, or Indigenous). Non-native path distance includes just the distance from Addis Ababa to their place of origin just before immigration. Native path distance is from Addis Ababa to the final destination. Native Hawaiians have a waypoint in the Pacific Ocean at Tahiti. Migration destination for calculating distance for enslaved Black Africans taken to North or South America is Senegal in West Africa.

## Supplementary Information

**Additional file 1 **Supplementary data consists of figures: **Figure S1-S7**, **Tables Table S1-S8**, data sets for the fulfilled and unknown 6ddps for complex βmys/MYPBC3 and MAPT/MYBPC3, and computer code. Data sets are contained in files 6ddpbmysMYBPC3.xls and 6ddpMAPTMYBPC3.xls. Computer code is in file 2d_cg.nb.

## Data Availability

All data generated or analyzed during this study are included in this published article and its supplementary information files.
