# Supplementary material for: Natural variant frequencies across domains from different sarcomere proteins cross-correlate to identify inter-protein contacts associated with cardiac muscle function and disease
Source: Mol Biomed. 2021 Nov 15;2:35. doi: 10.1186/s43556-021-00056-x (PMC8607394; doi:10.1186/s43556-021-00056-x)
Supplement: Supplementary file 1 — Additional file 1 Supplementary data consists of figures: Figure S1-S7, Tables Table S1-S8, data sets for the fulfilled and unknown 6ddps for complex βmys/MYPBC3 and MAPT/MYBPC3, and computer code. Data sets are contained in files 6ddpbmysMYBPC3.xls and 6ddpMAPTMYBPC3.xls. Computer code is in file 2d_cg.nb. [file 43556_2021_56_MOESM1_ESM.zip › Supplementary2.docx]

Supplementary Material

Natural variant frequencies across domains from different sarcomere proteins cross-correlate to identify inter-protein contacts associated with cardiac muscle function and disease

Thomas P. Burghardt

Department of Biochemistry and Molecular Biology

200 First St. SW

Mayo Clinic Rochester

Rochester, MN 55905

[burghardt@mayo.edu](mailto:burghardt@mayo.edu)

[https://orcid.org/0000-0003-3119-4074](https://orcid.org/0000-0003-3119-4074?lang=en)

Molecular Biomedicine

May 2021, Revised July 2021, Revised August 2021, Revised September 2021


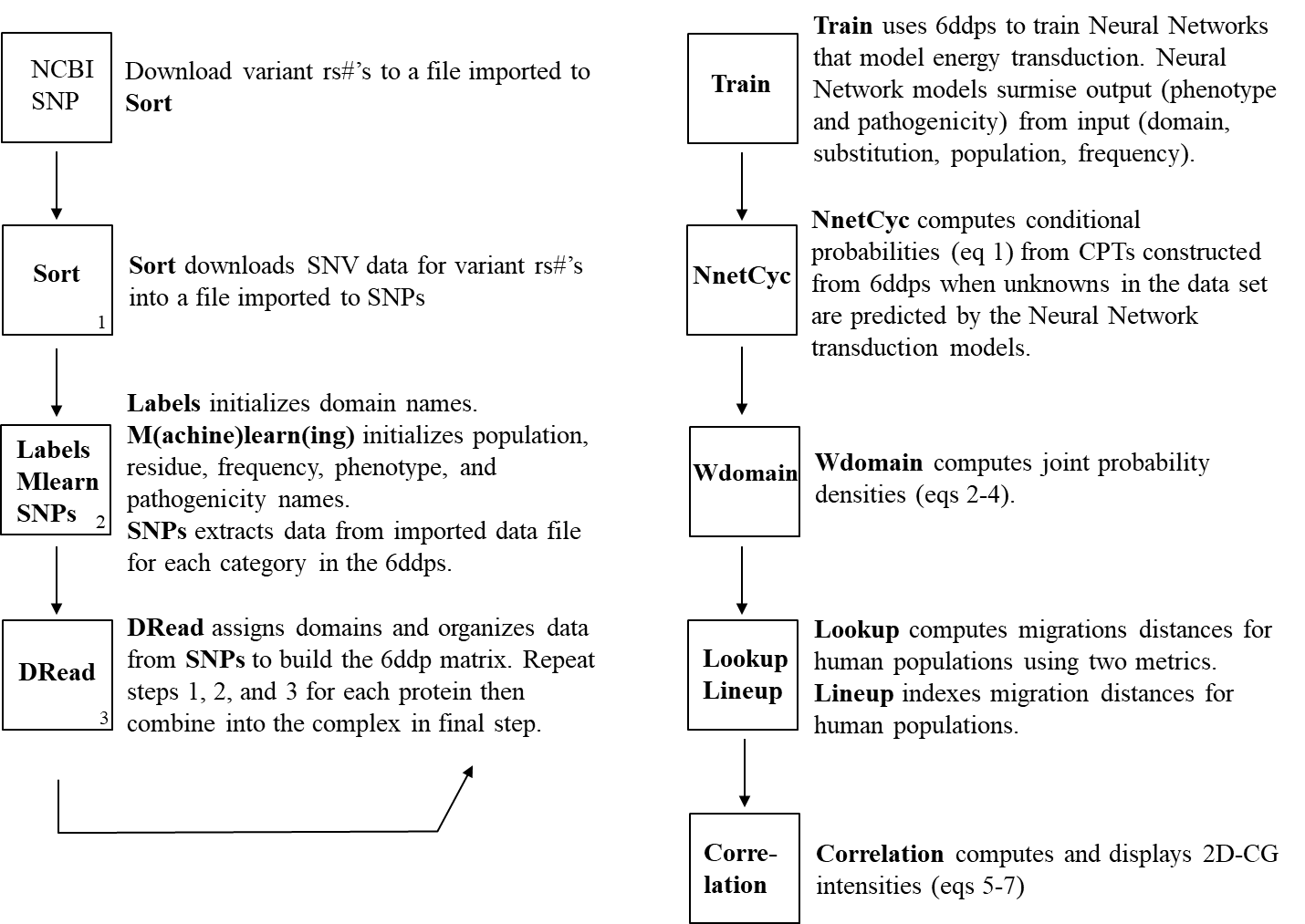


**Figure S1**. Using a browser, open the National Center for Bioinformatics (NCBI) home page, select the SNP database, and use “MYH7 missense_variant” as the search item. The search returns items saved to a text file that is imported to the Mathematica (Wolfram, Champaign, IL, USA) program Sort. Sort uses text search/extract tools to identify and collect the SNV reference numbers (rs#) into a list. Redundant rs# entries are merged. Each rs# corresponds to a single reference gene and nucleotide location but may involve various nucleotide substitutions. Mathematica programs in steps 1-3 generally need minor adjustments with each NCBI SNP data build. Other programs appearing in the figure are likewise coded in Mathematica. Source code is in **Supplementary** file 2d_cg.nb.


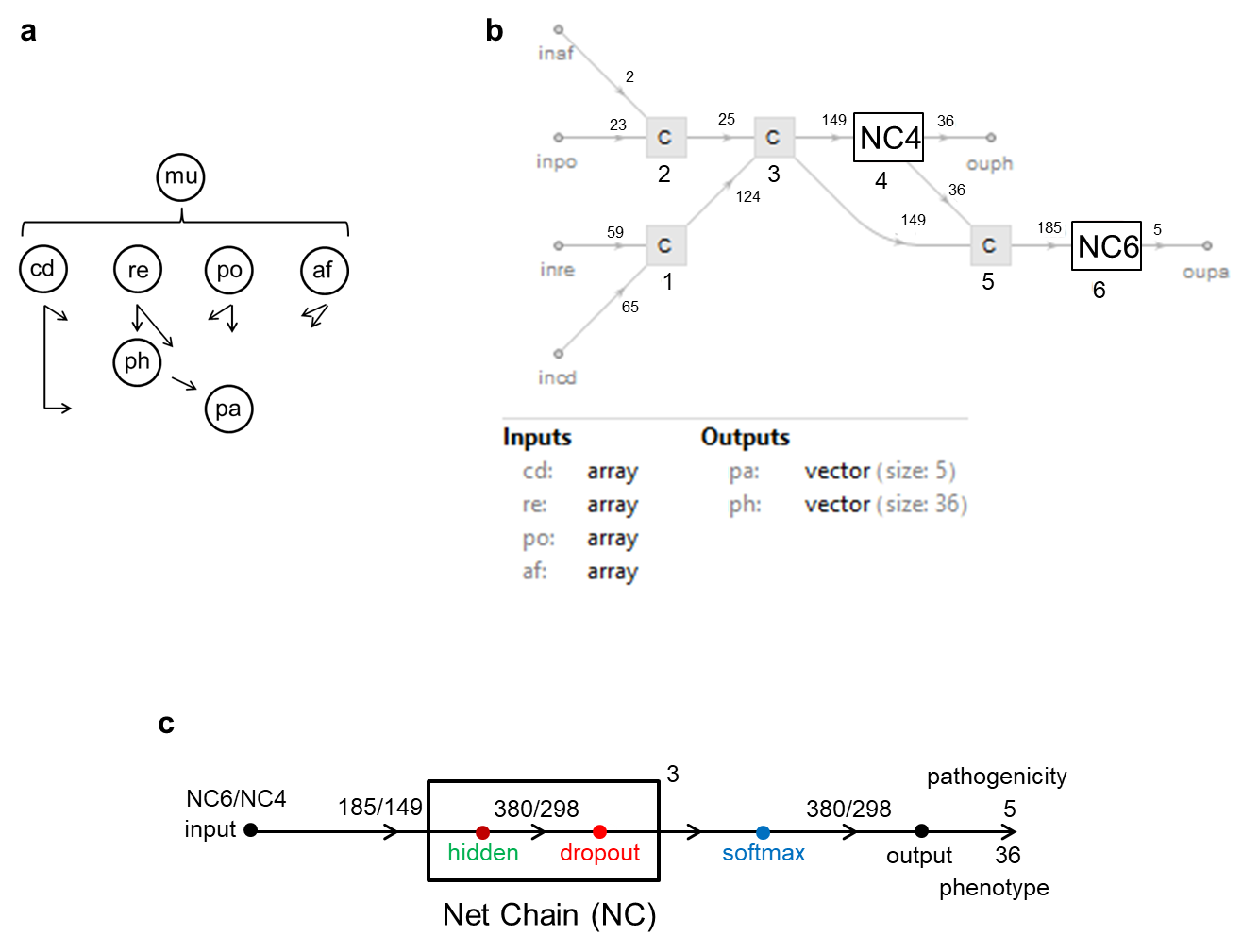


Figure S2. The neural network modeling structure/function influences from heart disease. a. Directed acyclic graph (DAG) denoting relationships among mutant (mu) domain location (cd), residue substitution (re), human population (po), allele frequency (af), phenotype (ph), and pathogenicity (pa) modeling the contraction mechanism with neural and Bayes networks. b. Feed forward neural net corresponding to the DAG model for contraction from Fig. S2a and relating inputs (prefix in) for the site of the SNV modification, residue substitution, human population, and allele frequency with outputs (prefix ou) for disease phenotype and pathogenicity. Components denoted by C (1-3, and 5) catenate input lists to Net Chains denoted with NC (components 4 and 6). Numbers near arrows in the lines input to or output from C and NC components are the number of nodes. Node number shown are for βmys/MYBPC3 in complex with 65 domains and 59 ref/sub pairs input to C1, and, 23 human population categories and 2 allele frequency categories input to C2. Output phenotypes and pathogenicities number 36 and 5, respectively. c. Net Chains are three connected hidden/dropout layers (superscripted 3) that output to a softmax layer conditioning output for digital classification of phenotype and pathogenicity. Numbers above the arrows in the horizontal line are nodes for NC6/NC4 in panel b. Node numbers are for the βmys/MYBPC3 complex.


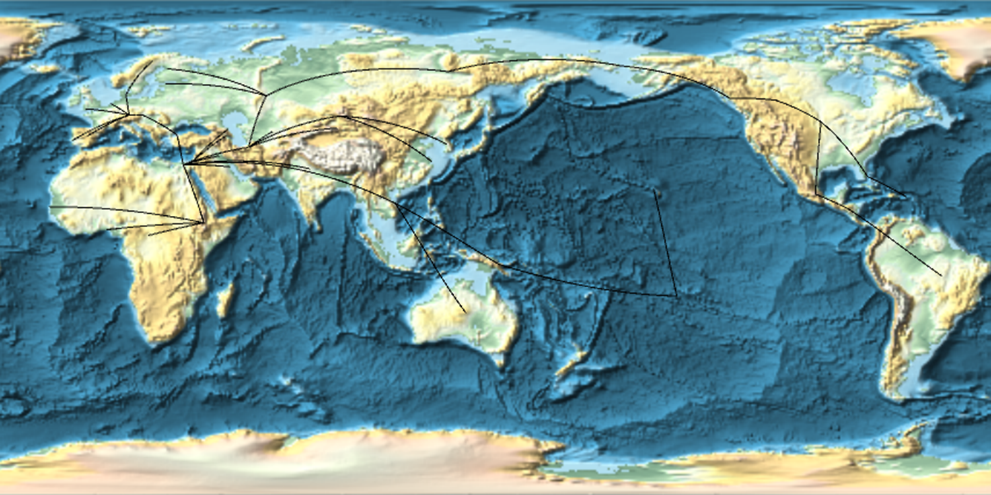


Figure S3. Migration pathways shown with black lines starting from Addis Ababa, Ethiopia and ending at points used to estimate migration distance for populations listed in Supplementary Tables S7 & S8. Migration to all destinations, excluding those ending in Africa, are via Cairo, Egypt.

**
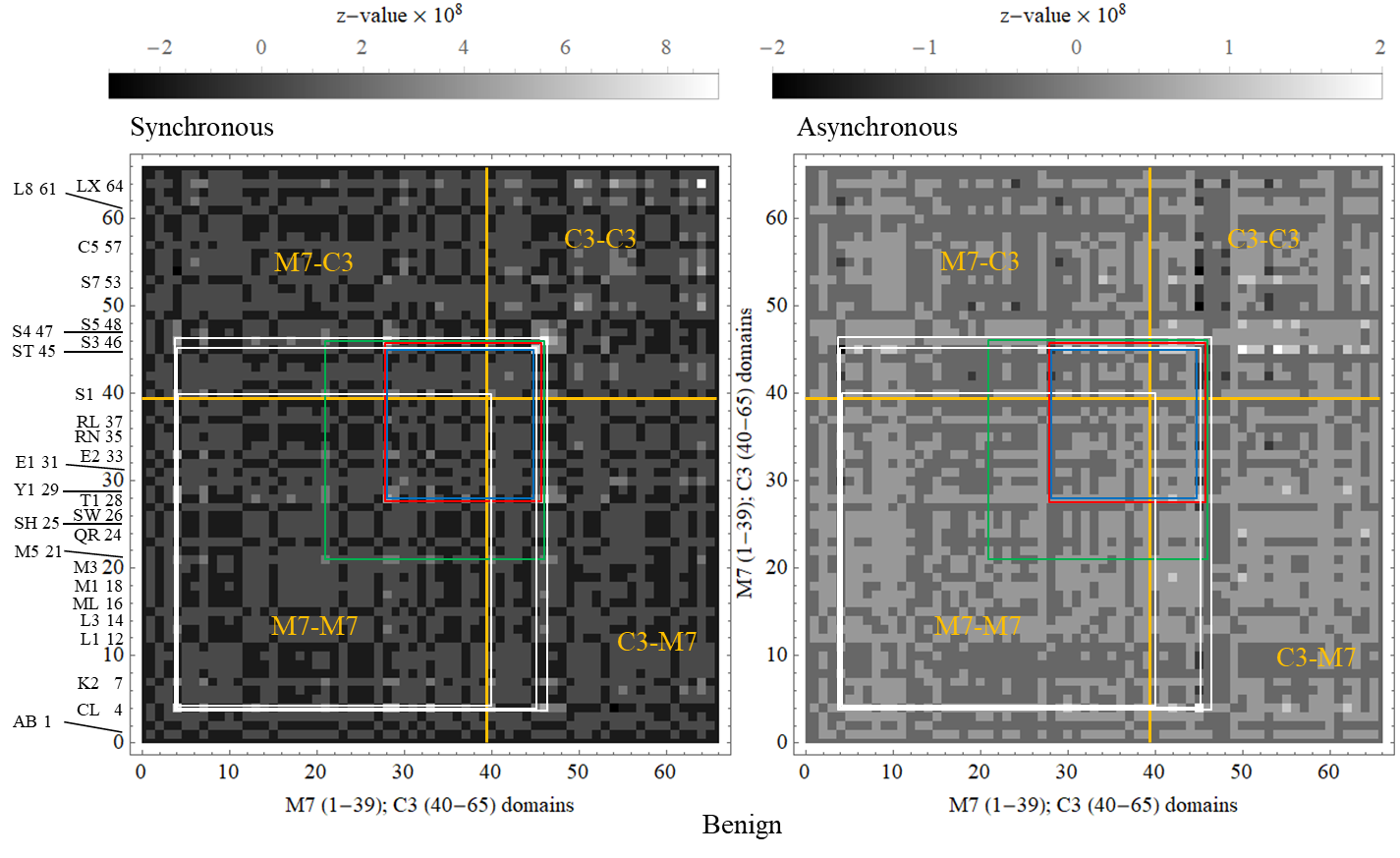
**

**Figure S4.** βmys/MYBPC3 complex correlation maps for benign outcomes implied by 2D-CG. Synchronous (left) or asynchronous (right) maps have axes identical for both dimensions representing βmys (M7 1-39) followed by MYBPC3 (C3 40-65) domains. Domain index is linked to its two-letter code and protein sequence in Supplementary **Table S1**. Two-letter codes for domains label some indices on the leftmost axis in the figure. Intensities (z-values) are indicated numerically by the grayscale. Regions defined by vertical and horizontal orange lines at the interface of pixels 39-40 separate intra-protein cross-peaks (within regions M7-M7 and C3-C3) from inter-protein cross-peaks (within regions M7-C3 and C3-M7). Intensity peaks along the diagonal in the synchronous map are autocorrelated probabilities for each domain. Correlation squares link the 6 most significant off-diagonal co-domain coordinates falling within the M7-C3 and C3-M7 regions. They are white, green, red, blue, for different βmys domains then repeating color sequence as needed.

**
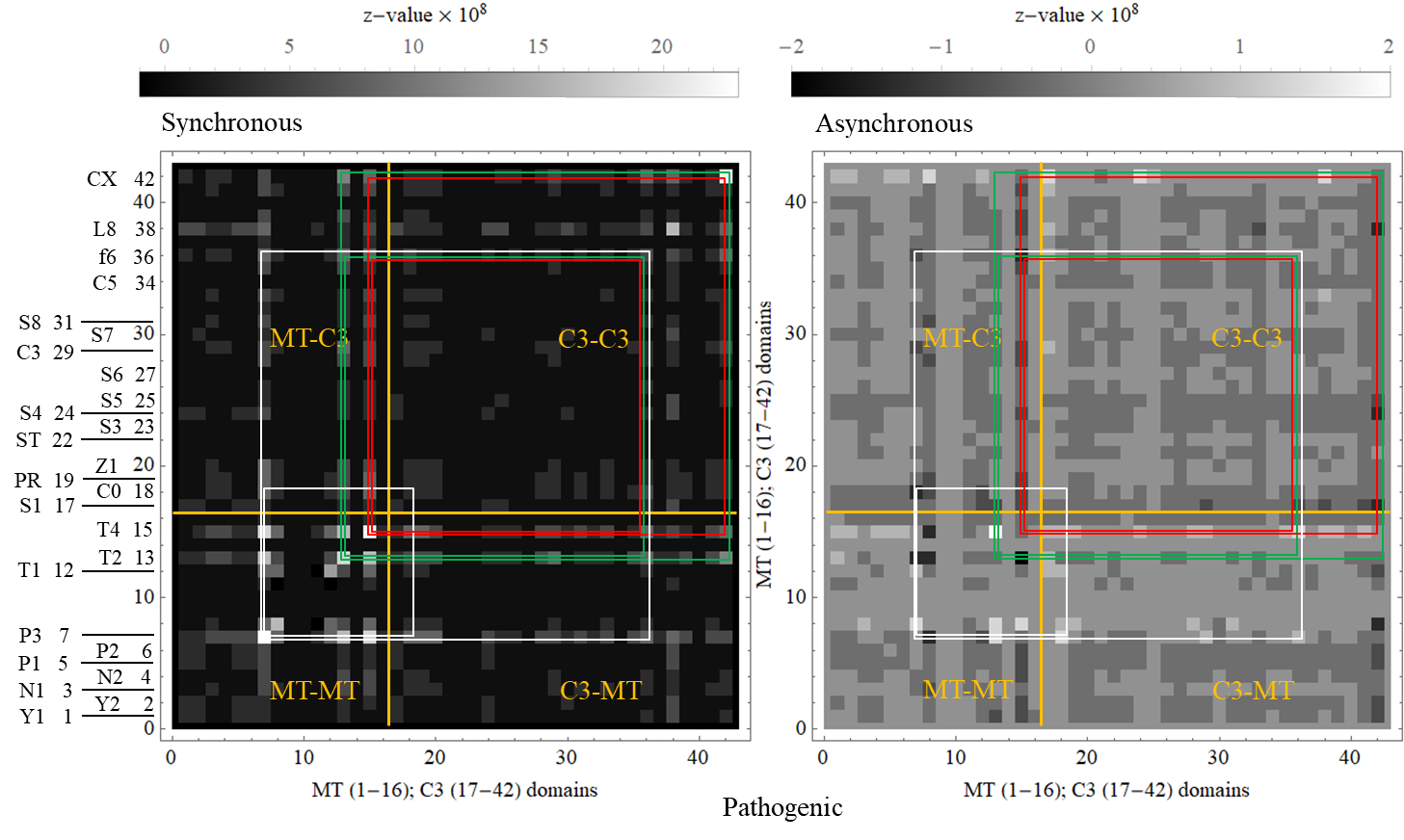
**

**Figure S5.** MAPT/MYBPC3 complex correlation maps for pathogenic outcomes implied by 2D-CG. Synchronous (left) or asynchronous (right) maps have axes identical for both dimensions representing MAPT (MT 1-16) followed by MYBPC3 (C3 17-42) domains. Domain index is linked to its two-letter code and protein sequence in Supplementary **Table S4**. Two-letter codes for domains label some indices on the leftmost axis in the figure. Intensities (z-values) are indicated numerically by the grayscale. Regions defined by vertical and horizontal orange lines at the interface of pixels 16-17 separate intra-protein cross-peaks (within regions MT-MT and C3-C3) from inter-protein cross-peaks (within regions MT-C3 and C3-MT). Intensity peaks along the diagonal in the synchronous map are autocorrelated probabilities for each domain. Correlation squares link the 6 most significant off-diagonal co-domain coordinates falling within the MT-C3 and C3-MT regions. They are white, green, red, blue, for different βmys domains then repeating color sequence as needed.

**
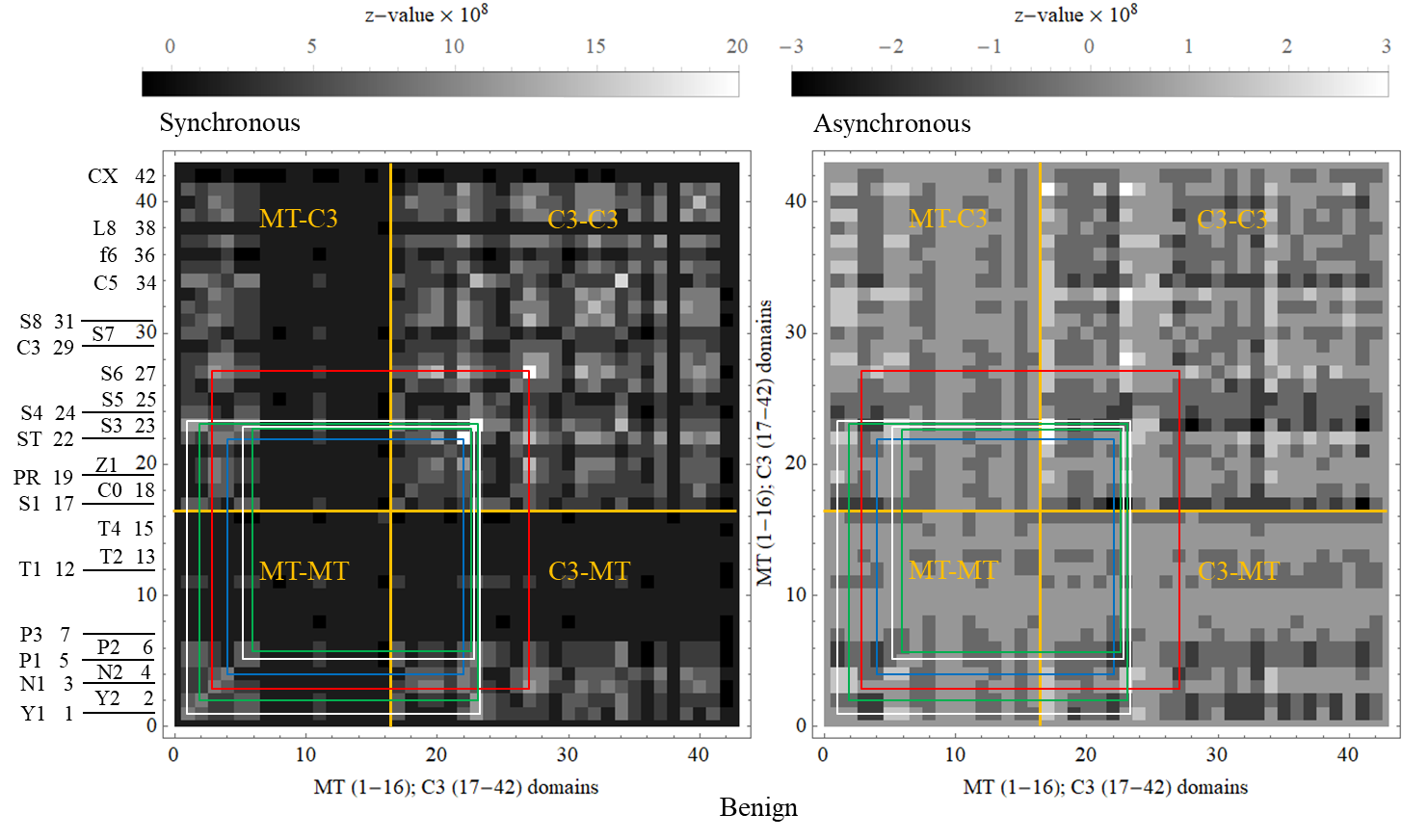
**

**Figure S6.** 2D correlation maps for complex MAPT/MYBPC3 with benign outcomes. Nomenclature is otherwise identical to Supplementary **Fig. S5**.

**
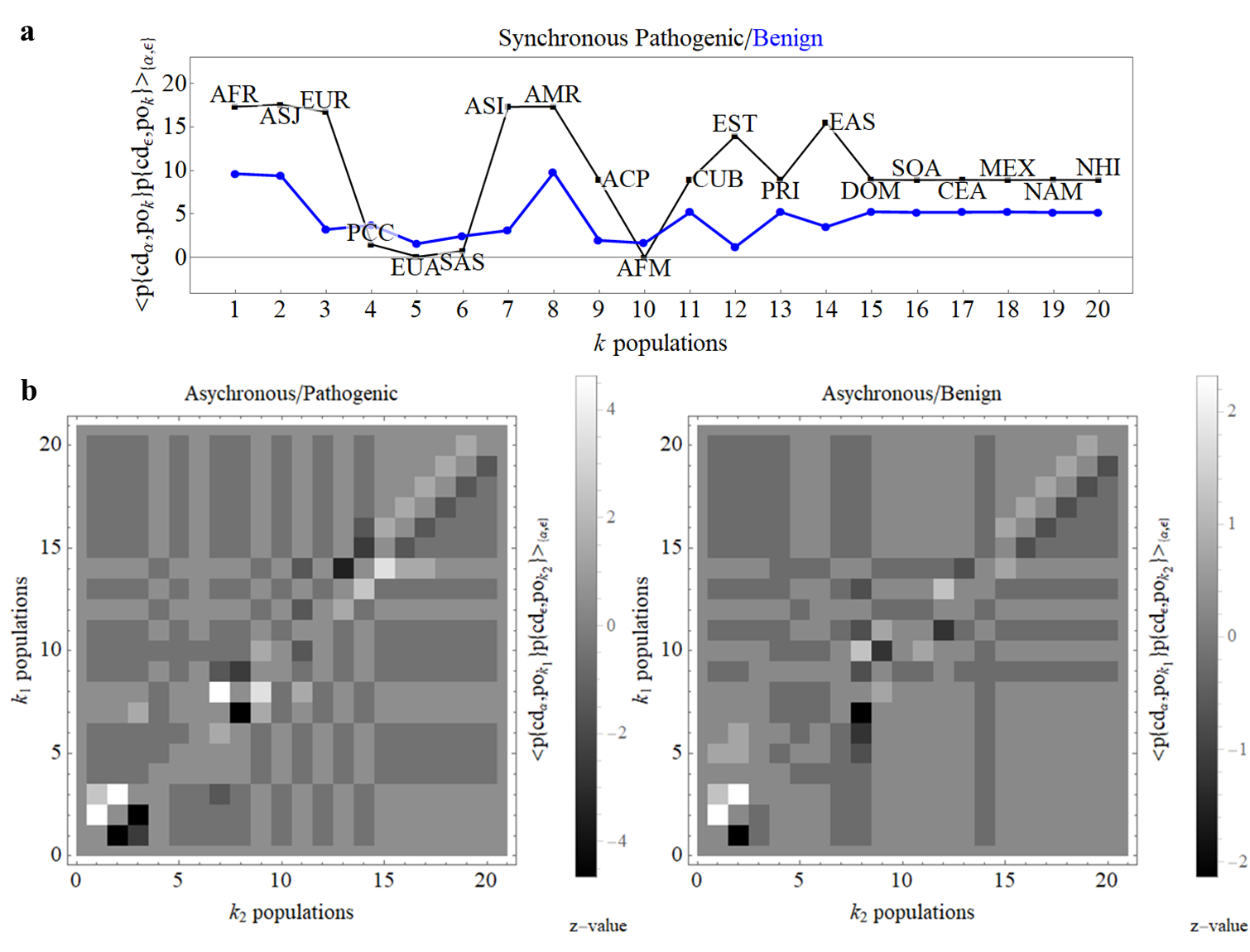
**

**Figure S7.** Population dependence for the 6 most significant co-domain interactions in the βmys/MYBPC3 complex using data from **Fig. 5** and **Fig. 6** for pathogenic and benign SNVs, and the quantities,

$<p\left\{ \mathrm{cd}_{\alpha},\mathrm{po}_{k} \right\}p\left\{ \mathrm{cd}_{\epsilon},\mathrm{po}_{k} \right\}>_{\left\{ \alpha,\epsilon\right\}}$ (S1)

or

$<p\left\{ \mathrm{cd}_{\alpha},\mathrm{po}_{k1} \right\} nf\left[ {cd}_{\alpha},{cd}_{\epsilon} \right] p\left\{ \mathrm{cd}_{\epsilon},\mathrm{po}_{k2} \right\}>_{\left\{ \alpha,\epsilon\right\}}$ (S2)

for synchronous (eq. S1) or asynchronous (eq. S2) correlations, <…>_{α,ε}_ implying averaging over the most significant co-domains (*cd_α_*,*cd_ε_*), p probabilities in eqs 3-4, and population *po_k_*. Asynchronous population dependence (eq. S2) involves population pairs (k_1_,k_2_) requiring two dimensions for display.

**Fig. S7a** has synchronous interactions for pathogenic (black) or benign (blue) SNVs. **Fig. S7b** has asynchronous interactions for pathogenic (left) or benign (right) SNVs. Population three letter code and index are indicated in **Fig. S7a**. Averaged quantities are defined in eqs. S1-S2 for the {α,ε} ≡{M7,C3} co-domains where pathogenic {α,ε} = {ML,C5}, {M3,C5}, {E1,C5}, {SW,L8}, {L3,L8}, and {M1,L8} or benign {α,ε} = {CL,S1}, {CL,ST}, {CL,S3}, {M5,S3}, {T1,ST}, and {T1,S3}.

**
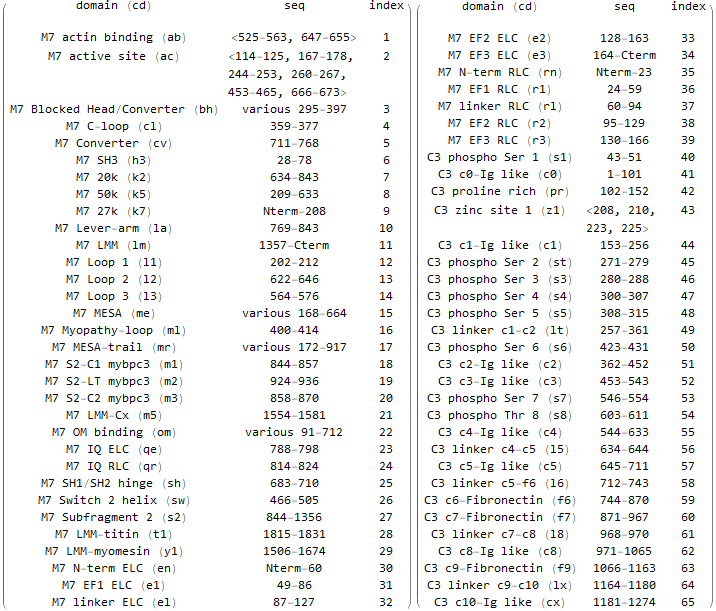
**

**Table S1.** Protein domain names, two letter codes (cd), protein sequence assignment (seq), and indexed numbering for domains in the complex βmys/MYBPC3. Domain names begin with M7 or C3 designating origin from βmys or MYBPC3, respectively. Myosin default domains 27k, 50k, and 20k refer to the tryptic proteolytic fragments from cleavage of the MHC sequence in Loop 1 at the active site and Loop 2 in the actin binding site [1]. OM binding (om) is the binding site for Omecamtiv Mecarbil [2]. Many domains are identified within the linearized overall protein structure in **Fig. 2**.


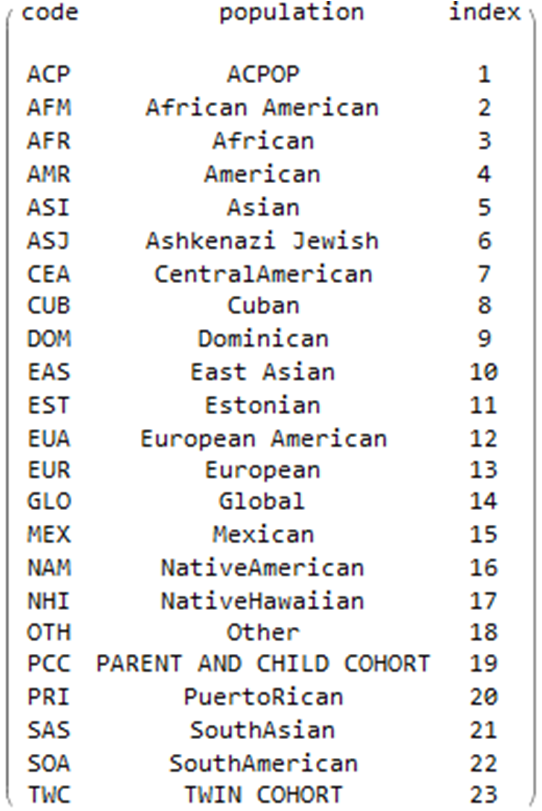


**Table S2**. Human population codes and descriptions pertaining to complex βmys/MYBPC3 from 1000Genomes, gnomAD-Genomes, TopMed, and other studies (see below) in the NCBI database pertaining to βmys and MYBPC3 SNP variants.

ACPOP (ACP) whole-genome sequenced control population study from Västerbotten County in northern Sweden; Dominican (DOM) Dominican Republic; Global (GLO) worldwide aggregate missense SNV data from sequencing studies that vary by SNV; Other (OTH) missense SNVs where a population category is not indicated; Parent and Child Cohort (PCC) the UK10K Avon Longitudinal Study of Parents and Children Variants; Twin Cohort (TWC) the UK10K Department of Twin Research and Genetic Epidemiology twin registry of 11,000 identical and non-identical twins between the ages of 16 and 85 years.


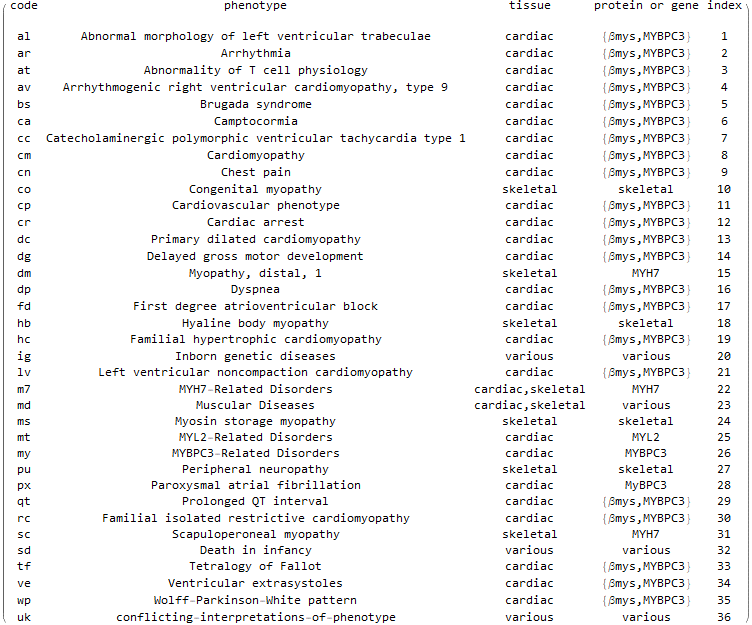


**Table S3**. Phenotype (ph) 2 letter codes, descriptive names, symptomatic tissues, protein or gene SNV sites, and numerical index pertaining to complex βmys/MYBPC3. Most phenotypes associate with cardiac disease (tissue column) from SNVs modifying either βmys or MYBPC3 (protein or gene column). Conflicting-interpretation-of-phenotype (code uk) implies no consensus phenotype from the database.


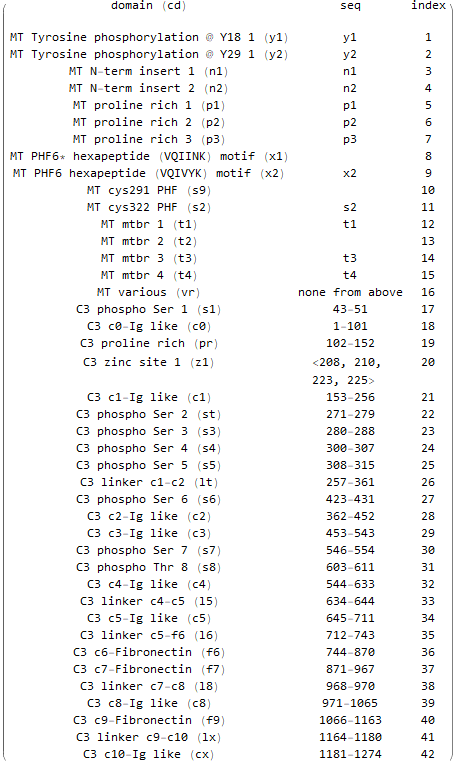


**Table S4.** Protein domain names, two letter codes (cd), protein sequence assignment (seq), and indexed numbering for domains in the complex MAPT/MYBPC3. Domain names begin with MT or C3 designating origin from MAPT or MYBPC3, respectively. MAPT sequence is not numerical because each isoform has a different sequence. Acronym mtbr is for MAPT microtubule binding repeat. Various domain (vr) includes every residue not falling into otherwise named domains. Domain vr is non-functional and ignored in the analysis tracing co-domains in complex MAPT/MYBPC3. All named domains are shown in **Fig. 2**.


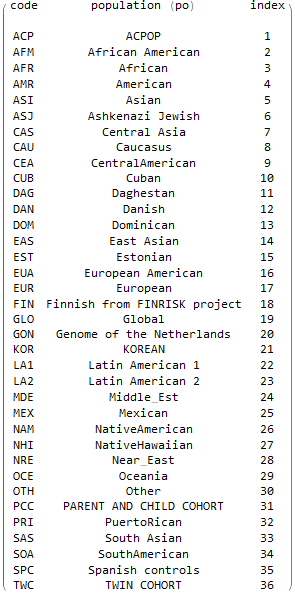


**Table S5**. Human population codes and descriptions pertaining to complex MAPT/MYBPC3 from 1000Genomes, gnomAD-Genomes, TopMed, and other studies (see below) in the NCBI database pertaining to MAPT and MYBPC3 SNP variants.

ACPOP (ACP) whole-genome sequenced control population study from Västerbotten County in northern Sweden; Dominican (DOM) Dominican Republic; Global (GLO) worldwide aggregate missense SNV data from sequencing studies that vary by SNV; Latin American 1 (LA1) Latin American individuals with Afro-Caribbean ancestry; Latin American 2 (LA2) Latin American individuals with mostly European and Native American Ancestry; Other (OTH) missense SNVs where a population category is not indicated; Parent and Child Cohort (PCC) the UK10K Avon Longitudinal Study of Parents and Children Variants; Spanish controls (SPC) Medical Genome Project healthy controls from Spanish population; Twin Cohort (TWC) the UK10K Department of Twin Research and Genetic Epidemiology.


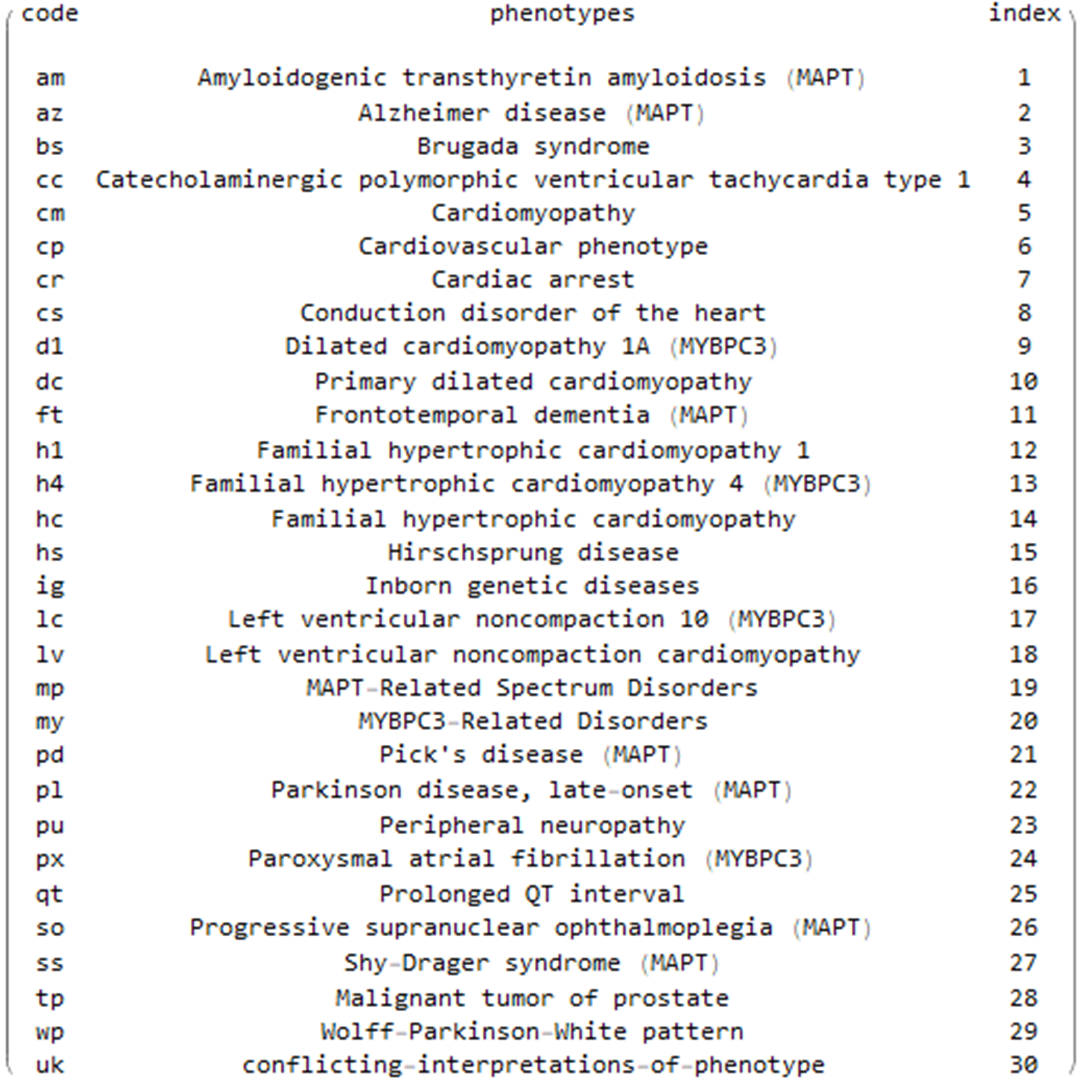


**Table S6**. Phenotype (ph) with 2 letter codes pertaining to complex MAPT/MYBPC3. Phenotypes associated exclusively with MAPT or MYBPC3 are indicated with the gene name in parenthesis otherwise they are associated with both MAPT and MYBPC3 modifications. Conflicting-interpretation-of-phenotype (code uk) implies no consensus phenotype from the database.


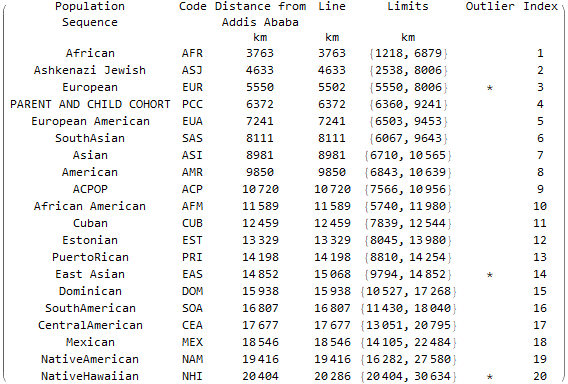

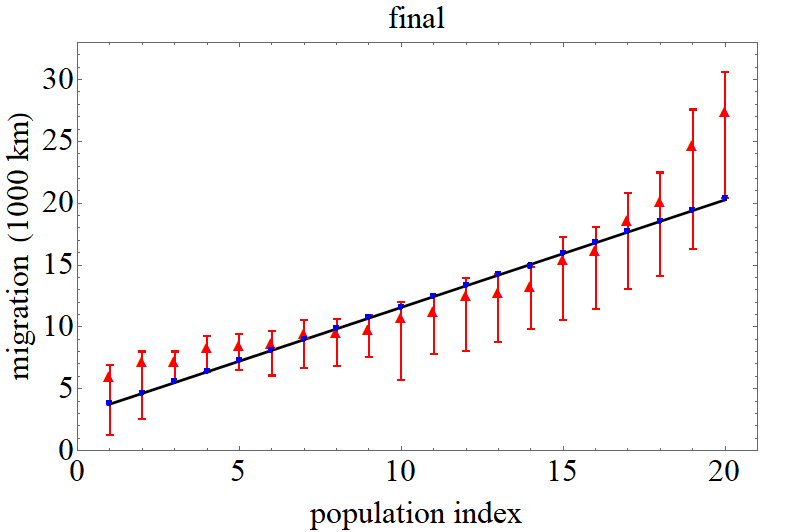


**Table S7**. Migration distance vs human population index for the Supplementary **Table S2** data set pertaining to complex βmys/MYBPC3 in tabular (top) and graphical (bottom) forms. **Top**. Populations (column 1) listed are in a linear relationship with migration distance (column 3). The migration distance is a proxy for genetic divergence variation where divergence decreases with distance. Fitted line (column 4), computed as described in the text, closely follows migration distances (column 3) and falls within limits (column 5) except for outliers identified with the asterisk in column 6. We use the population index (column 7) to identify populations in graphical presentations like that in the bottom panel. **Bottom**. Blue line indicates a best estimate for the linear relationship of listed populations with migration distance. Red vertical bars at each triangle show minimum limits needed to fulfill the linear estimate as described in the text. Red triangles indicate migration distances falling within the red vertical bars best fitted by the blue line.


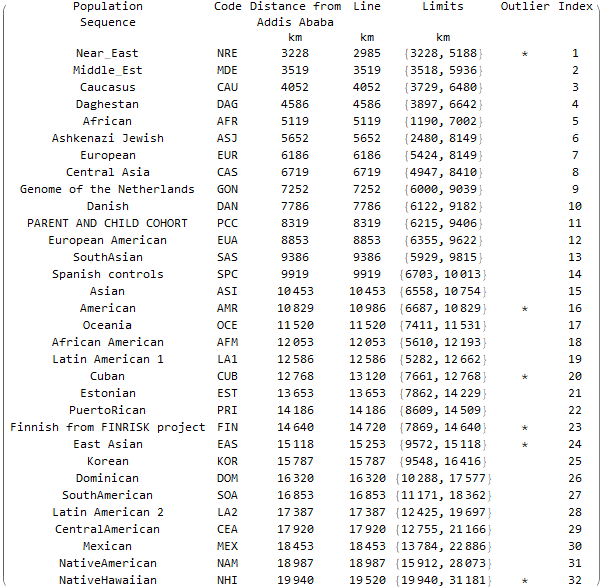


**a**

**Table S8.** Migration distance vs human population index for the Supplementary **Table S5** data set pertaining to complex MAPT/MYBPC3 in tabular (**Table S8a**) and graphical (**Table S8b**) forms. **a**. Populations (column 1) listed are in a linear relationship with migration distance (column 3). The migration distance is a proxy for genetic diversity variation where diversity decreases with distance. Fitted line (column 4), computed as described in the text, closely follows migration distances (column 3) and falls within limits (column 5) except for outliers identified with the asterisk in column 6. We use the population index (column 7) to identify populations in graphical presentations like that in **Table S8b** below.


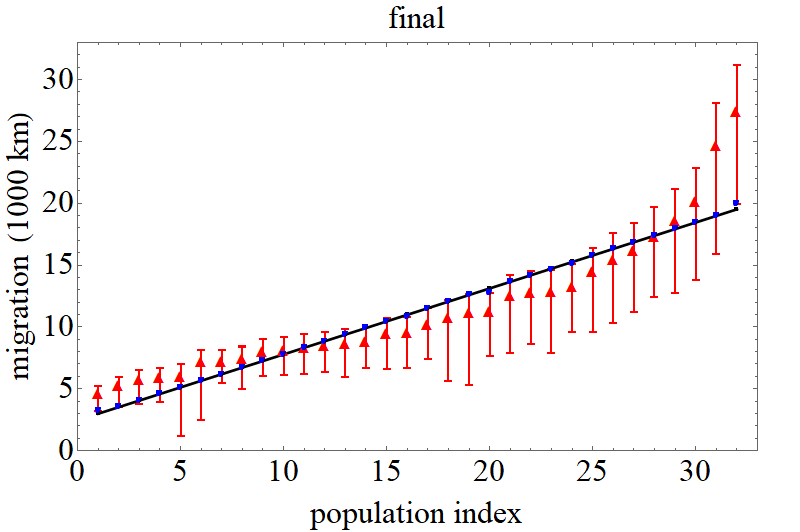


**b**

**b**. Blue line indicates a best estimate for the linear relationship of listed populations with migration distance. Red vertical bars at each triangle show minimum limits needed to fulfill the linear estimate as described in the text. Red triangles indicate migration distances falling within the red vertical bars best fitted by the blue line.

**Data Sets**

1. Name: 6ddp complex βmys/MYPBC3

Caption: Fulfilled and unknown 6 dimensional data points (6ddp) for complex βmys/MYPBC3

Description: Fulfilled and unknown 6 dimensional data points (6ddp) for complex βmys/MYPBC3 containing mutation site, residue substitution, phenotype, and pathogenicity.

File name: 6ddpbmysMYBPC3.xls

2. Name:6ddp complex MAPT/MYBPC3

Caption: Fulfilled and unknown 6 dimensional data points (6ddp) for complex MAPT/MYBPC3

Description: Fulfilled and unknown 6 dimensional data points (6ddp) for complex MAPT/MYBPC3 containing mutation site, residue substitution, phenotype, and pathogenicity.

File name: 6ddpMAPTMYBPC3.xls

References

1. Balint M, Sreter FA, Wolf I, Nagy B, Gergely J. The substructure of heavy meromyosin. The effect of Ca 2+ and Mg 2+ on the tryptic fragmentation of heavy meromyosin. *J Biol Chem.* 1975;250:6168-6177.doi:10.1016/S0021-9258(19)41173-3.

2. Liu Y, White HD, Belknap B, Winkelmann DA, Forgacs E. Omecamtiv mecarbil modulates the kinetic and motile properties of porcine β-cardiac myosin. *Biochemistry.* 2015;54(10):1963-1975.doi:10.1021/bi5015166.
